# Proteomic analysis of plasma exosomes from Cystic Echinococcosis patients provides *in vivo* support for distinct immune response profiles in active *vs* inactive infection and suggests potential biomarkers

**DOI:** 10.1371/journal.pntd.0008586

**Published:** 2020-10-05

**Authors:** Federica Fratini, F. Tamarozzi, G. Macchia, L. Bertuccini, M. Mariconti, C. Birago, A. Iriarte, E. Brunetti, CM. Cretu, O. Akhan, M. Siles-Lucas, A. Díaz, Adriano Casulli

**Affiliations:** 1 Proteomics Core Facility, Istituto Superiore di Sanità(ISS), Rome, Italy; 2 Who Collaborating Centre for Epidemiology, Detection and Control of Cystic and Alveolar Echinococcosis, Department of Infectious, Diseases (DMI), ISS, Rome, Italy; 3 Electron Microscopy Core Facility, ISS, Rome, Italy; 4 Department of Clinical Surgical Diagnostic and Paediatric Sciences, University of Pavia, Pavia, Italy; 5 Department of Infectious, Diseases (DMI), ISS, Rome, Italy; 6 Laboratorio de Biología Computacional, Dpto. de Desarrollo Biotecnológico, Instituto de Higiene, Facultad de Medicina, Universidad de la República, Montevideo, Uruguay; 7 University of Medicine and Pharmacy, Colentina Clinical Hospital—Parasitology, Bucharest, Romania; 8 Faculty of Medicine, Hacettepe University, Ankara, Turkey; 9 Instituto de Recursos Naturales y Agrobiologı´a de Salamanca (IRNASA-CSIC), Salamanca, Spain; 10 Área Inmunología, Departamento de Biociencias, Facultad de Química, Universidad de la República, Montevideo, Uruguay; 11 European Reference Laboratory for Parasites (EURLP), DMI, ISS, Rome, Italy; Universidad Peruana Cayetano Heredia, PERU

## Abstract

The reference diagnostic method of human abdominal Cystic Echinococcosis (CE) is imaging, particularly ultrasound, supported by serology when imaging is inconclusive. However, current diagnostic tools are neither optimal nor widely available. The availability of a test detecting circulating biomarkers would considerably improve CE diagnosis and cyst staging (active vs inactive), as well as treatments and follow-up of patients. Exosomes are extracellular vesicles involved in intercellular communication, including immune system responses, and are a recognized source of biomarkers. With the aim of identifying potential biomarkers, plasma pools from patients infected by active or inactive CE, as well as from control subjects, were processed to isolate exosomes for proteomic label-free quantitative analysis. Results were statistically processed and subjected to bioinformatics analysis to define distinct features associated with parasite viability. First, a few parasite proteins were identified that were specifically associated with either active or inactive CE, which represent potential biomarkers to be validated in further studies. Second, numerous identified proteins of human origin were common to active and inactive CE, confirming an overlap of several immune response pathways. However, a subset of human proteins specific to either active or inactive CE, and central in the respective protein-protein interaction networks, were identified. These include the Src family kinases Src and Lyn, and the immune-suppressive cytokine TGF-β in active CE, and Cdc42 in inactive CE. The Src and Lyn Kinases were confirmed as potential markers of active CE in totally independent plasma pools. In addition, insights were obtained on immune response profiles: largely consistent with previous evidence, our observations hint to a Th1/Th2/regulatory immune environment in patients with active CE and a Th1/inflammatory environment with a component of the wound healing response in the presence of inactive CE. Of note, our results were obtained for the first time from the analysis of samples obtained *in vivo* from a well-characterized, large cohort of human subjects.

## Author summary

Exosomes represent an important way of cell-cell communication. Their release into the bloodstream is similar to sending messages in a bottle that can safely reach target tissues and cells. In disease contexts, these vesicles provide a suitable source of markers of pathogenesis or carcinogenesis, as well as of the immune response and the host-pathogen interplay. In cystic echinococcosis (CE), the availability of a marker-based blood test would represent an extraordinary improvement for disease diagnosis, staging and follow-up. The active search for CE carriers, realized by ultrasound surveys of around 25,000 individuals in the HERACLES project, allowed the implementation of a proteomic biomarker discovery analysis based on plasma exosomes from CE patients. The identification of parasite proteins likely within host exosomes supports the occurrence of this important exchange of information through exosomes and suggests possible molecular markers to be further validated. Finally, our study provides complementary and supporting information to previous evidence on the types of response mounted by the immune system in the presence of active or inactive CE, exploiting data obtained *in vivo*, from a large, well-characterized cohort of subjects.

## Introduction

Cystic echinococcosis (CE) is a parasitic zoonosis caused by the cystic larval stage (metacestode) of the dog tapeworm *Echinococcus granulosus sensu lato*. The global burden of human CE has been estimated in more than 1 million people infected, with over 1 million DALYs lost every year, when accounting for underreporting [1]. The WHO has enlisted CE among the neglected zoonoses prioritized for control efforts [2]. The parasite is transmitted between canid definitive hosts (mainly dogs) and intermediate hosts (mainly livestock, in particular sheep) through the fecal-oral route. Humans are dead-end hosts acquiring the infection by ingesting parasite eggs shed through infected dog feces. CE is globally distributed, in particular in rural areas where livestock breeding practices maintain the life cycle of the parasite [3]. Echinococcal cysts may develop in humans in any organ or tissue, most commonly affecting the liver and the lungs [4]. Most infected people, especially those harboring abdominal CE, are asymptomatic or pauci-symptomatic for a long time or even lifelong. On the contrary, up to 10% of infections may cause serious, disabling or even fatal disease, especially in case of complications, dissemination, or involvement of organs such as the central nervous system or the bones [4]. CE cysts pass through different stages, spontaneously or as the consequence of non-surgical treatments. The current WHO-IWGE (Informal Working Group on Echinococcosis) classification of CE cysts is based on ultrasound and encompasses six stages: CE1, CE2, CE3b (active cysts); CE3a (transitional cysts), and CE4, CE5 (inactive cysts) [5, 6]. Ultrasound (US) is the reference diagnostic tool for staging abdominal CE, supported by serology when imaging is inconclusive [5]. However, US is an operator-dependent exam and US machines and/or expertise to operate them are seldom available in rural endemic areas. Serology is currently not standardized, is burdened by unsatisfactory performances [7], and does not solve questions relative to the biological viability of the metacestode. A recent study, reporting a three years follow up of 27 cytokines and IgG antibodies’ production in the serum of four CE patients, highlighted the difficulties in using these molecules as markers of cyst activity [8]. The availability of a test detecting reliable circulating biomarkers would considerably improve the diagnosis of human CE and assessment of cyst viability, with great impact on the management of patients. Such a test, ideally in the format of a point-of-care test, would avoid costs due to misdiagnoses [9] and dramatically reduce costs associated with the mismanagement and overtreatment of inactive cysts and with imaging-based long term follow-up [10].

Recently, there has been a growing interest in the study of extracellular vesicles (EVs). Body fluids comprise a variety of EVs secreted from different tissues and cell types. The release of vesicles with a double-layered lipid membrane is a highly conserved biological event in prokaryotes and eukaryotes, appearing as a common process shared by virtually all cell types. Among EVs, exosomes (EXO) are nano-sized vesicles (30–150 nm) generated within endosomal multivesicular bodies and secreted when these compartments fuse with the plasma membrane. Proposed to mediate cell-cell communication in patho-physiological conditions, currently EXO represent sensible biomedical research targets, extensively investigated for biomarker identification, for understanding cell communication in various contexts including metastatic processes and the host-pathogen interplay, or as drug delivery vehicles [11–14]. In pathogen infections, EXO have been reported as crucial messengers in the balance of infection processes, likely involved in the modulation of multiple signalling processes [15]. In particular, several recent studies have reported EXO exchange in helminth infections [16, 17]. A higher release of EXO has been widely reported by cells subjected to any kind of stress. The biological advantage of using EXO for cell–cell communication, under physiological or pathological conditions [18, 19], stems from their complex and regulated endosomal cargo composition and trafficking, which allows for stringent controls over the communication processes [14, 20]. Further, by increasing their stabilization and protecting them from degradation, EXO enable molecules, such as nuclei acids and proteins, to evade immunosurveillance mechanisms, and prevent events such as apoptosis or macrophage phagocytosis [21, 22]. The EXO cargo, which reflects their origin and target cells, as well as their trafficking, is highly regulated by several energy-dependent steps [23]. EXO may dock at the plasma membrane of the target cell and activate intracellular signaling by ligand-receptor interaction, or releasing their content into the cytoplasm of the recipient cells after being internalized by phagocytosis, or receptor-/raft-mediated endocytosis, or upon direct fusing with the membrane [12, 24]. The membrane structures of these vesicles, almost shared among all cells and species, suggests an exchange among inter-specie cells followed by the exchange among intra-specie cells. Thus, EXO delivery into the bloodstream can be described as analogous to sending messages in a bottle: every cell spreads numerous messenger molecules directed to target cells in an extremely sensitive and complex cell language, through protective vesicle trafficking.

One of the main goals and greatest challenges of clinical proteomics is the identification of potential plasma protein markers and possible target signalling involved in the establishment of a disease. Blood is easily accessible and can be described as the most comprehensive human proteome, potentially informative on almost any disease state. However, its comprehensiveness is counterbalanced by the complexity and high dynamic range of its protein mixture, with very abundant proteins interfering with the proteomic analysis. In this study, we investigate EXO isolated from human plasma of CE infected patients and control subjects. The aims of the study were to identify potential blood biomarkers within a “cleaner” and easier sub-proteome than blood, and to better understand the host-parasite interplay, which allows the masterful immuno-modulation and mutual coexistence, characteristic of helminth infections [17, 25]. Despite a wide use of biochemical techniques and isolation kits [26], most strategies to isolate EXO from plasma suffer from co-purifying EV sub-types (microvesicles, EXO and other protein aggregates) leading to confusion and misinterpretation of results [27, 28]. Importantly, most of available protocols come from studies where EXO are isolated from supernatants of in-vitro cell cultures or biological fluids less complex and less viscous than plasma [22]. Therefore, we set up a suitable method for a specific enrichment of EXO from human plasma and applied it to analyse the large and well characterized cohort of CE patient plasma samples collected within the framework of HERACLES project surveys in Italy, Turkey and Romania in 2014–2015 [29, 30].

## Materials and methods

### Study pipeline

**Fig 1:**
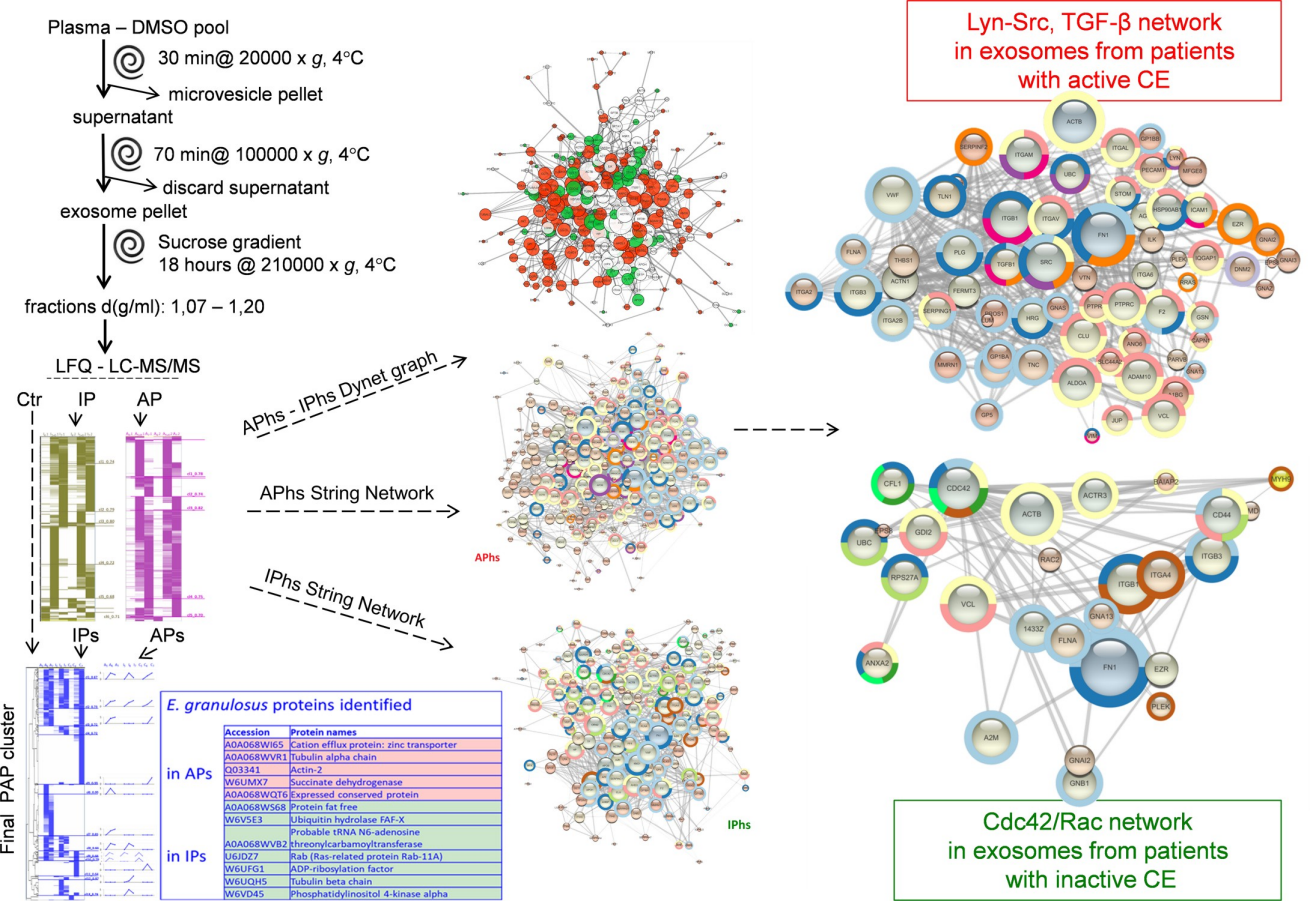
Study Pipeline. Plasma samples preserved in the presence of DMSO were pooled as described in Materials and Methods. Exosome pellets were obtained by differential centrifugation and removal of contaminant plasma proteins was improved by the use of sucrose gradient. Density fractions in the range (1,07–1,20 ± 0.02) g/ml were further processed for the label-free quantitative (LFQ) proteomics analyses by Liquid-Chromatography Tandem Mass Spectrometry (LC-MS/MS). Identified proteins quantified in at least two replicas (AP, IP) were submitted to hierarchical clustering, and those with Protein Abundance Profiles (PAPs) showing Pearson correlation R ≥ 0.7 (p ≤ 0.05) were selected (APs, IPs) for complete clustering, which included non-filtered control sample proteins (Ctr). Potential biomarkers belonging to E. *granulosus* were specifically identified in clusters of APs or IPs. Human proteins in APs and IPs (APhs, IPhs) were analyzed to obtain the interaction networks of enriched GO Biological Process and Reactome Pathways for each of APhs and IPhs (String Networks); a graph overlying both APhs and IPhs (Dynet graph) was built, which helped distinguishing between shared and specific proteins among the most central nodes. By analysing these outcomes, we could identify central proteins specifically enriched in exosomes from active or inactive CE, such as SFKs and TGF-β in active CE and Cdc42/Rac in inactive CE.

In this study we setup a suitable method to enrich and analyse exosomes from human plasma (**Fig 1**). On the basis of previous studies [31, 32] and data collected in our laboratory, we defined an experimental design for EXO preparation based on a pool strategy of almost 25 individual samples per pool (ca. 25 ml each), and analysed two independent pools for active CE samples, three for inactive CE and two for controls (**Table 1**). All plasma samples used for proteomic experiments were centrifuged at 10,000 x g before being stored at -80°C, in the presence of 5% v/v dimethylsulphoxide (plasma-DMSO). To efficiently separate EXO (30–150 nm) from microvesicles (MV; 100–1000 nm) two other sequential centrifugation steps, in fixed angle rotors, were performed. The first, to eliminate MV, was performed at 20,000 x g for 30 minutes, and the second, to collect EXO, was performed at 100,000 x g for 70 minutes, followed by a pellet wash in phosphate saline buffer (PBS) in the same conditions. The final pellet was loaded onto the bottom of a 5 ml sucrose gradient designed for the concentration of density fraction in the range of 1.10–1.16 g/ml. Fractions in the density range 1.07–1.20 ± 0.02 g/ml were analysed by label free quantitative (LFQ) mass spectrometry. The Protein Abundance Profiles (PAPs) of active CE pools (AP), inactive CE pools (IP) and control pools (Ctr) were calculated as described [33]. Proteins identified in at least two replicas and reporting PAP Pearson correlation values R ≥ 0.7 (p ≤ 0.05) were selected among AP and IP (APs, IPs). No analogous filtering was applied on proteins identified in control samples. The PAP median values were used to cluster all samples together (final PAP cluster). The *E*. *granulosus* proteins identified fell precisely either in the clusters of APs or IPs and constitute potential markers to be further validated. Human proteins in APs and IPs (APhs, IPhs) were analysed by Cytoscape platform using the String and Dynet applications to obtain and compare the protein-protein interaction networks and the enriched GO Biological Process and Reactome Pathways. This allowed distinguishing between shared proteins and group-specific ones within the set of proteins acting as central nodes. By analysing these outcomes, we could identify central proteins specifically enriched in EXO from patients with active or inactive CE, which suggest different pathways in the relative immune responses and potential markers of metacestode viability.

**Table 1:** Description of pool samples.

** **	**Plasma-DMSO pools for proteomics**				Plasma pools for WB*	
**Inactive CE pools (IP)**	**Cyst stage**	pool 1i	pool 2i	pool 3i	**Cyst stage**	pool WBi
		25 samples, 28 ml	25 samples, 27 ml	26 samples, 27 ml		48 samples, 30 ml
** **	CE4	12	13	13	CE4	19
**76 Total samples:**	CE4/CE4	4	4	4	CE4/CE4	8
66% Pavia Hospital	CE4+PSC	1			CE4+PSC	1
20% Turkey	CE4/CE4/CE4/CE4		0.5	0.5	CE4/CE4/CE4/CE4	1
14% Romania	CE5	6	4	5	CE5	14
	CE5/CE5		1	1	CE5+PSC	3
	CE5+PSC		1.5	1.5	CE5/CE5/CE5	1
	CE5/CE5/CE5	1			CE5/CE4/CE5	1
	CE5/CE4/CE5	1				
			pool 2a	pool 3a		pool WBa
**Active CE pools (AP)**			25 samples, 26 ml	23 samples, 25 ml		25 samples, 30 ml
	CE1		3	4	CE1	1
**46 Total samples:**	CE2		3	3	CE2	1
56% Pavia Hospital	CE3b		13	12	CE3b	20
31% Turkey	CE1/CE1		2	2	CE3b/CE2/CE1/CE1	1
13% Romania	CE1/CE2		1	1	CE4/CE3b/CE3b	1
	CE3b/CE4		1		CE4/CE3b/CE3b	2
	CE3b/CE2/CE1/CE1		1			
	CE4/CE3b/CE3b		1	1		
**Control pools (Ctr)**	**70 Total samples:** 80% Turkey; 20% Pavia Hospital. C1 = 25ml; C2 = 50ml.				* all CE samples were from Pavia Hospital while control samples (30 ml) from Turkey	

#### Ethics statement

All samples were collected upon written informed consent to the use of blood for research purposes. Ethical approval for the study was granted by the ethics committees of the Colentina Teaching Hospital (Bucharest, Romania) approved on 04/03/2014, Hacettepe University Hospital (Ankara, Turkey) N.16969557642, and IRCCS San Matteo Hospital Foundation (Pavia, Italy) N. 20150027307.

Blood used to set up the exosome preparation protocol was collected from healthy donors, kindly provided by Professor G. Girelli, University of Rome “La Sapienza”, Rome, Italy.

All samples were anonymized before analysis.

#### Sample collection and classification

Samples from individuals with CE were collected in the referral centres for CE of San Matteo Hospital Foundation, Pavia, Italy; Hacettepe University Hospital, Ankara, Turkey; and Colentina Clinical Hospital, Bucharest, Romania, as well as during the ultrasound-based population screenings for CE carried out in different sites of Romania and Turkey in 2014–2015 as part of the European project “HERACLES” [29, 34]. All samples were registered in the European Register of Cystic Echinococcosis (ERCE) as already described [35, 36] and stored in the EchinoBiobank at the IRNASA-CSIC in Salamanca, Spain, or directly shipped to the WHO Collaborating Centre for Epidemiology, Detection and Control of Cystic and Alveolar Echinococcosis at the Department of Infectious Diseases of Istituto Superiore di Sanità in Rome, Italy.

CE affected patients were identified using ultrasound on the basis of pathognomonic signs by clinicians experienced in the ultrasound diagnosis of CE. CE cysts were staged according to the WHO-IWGE classification [5]. Briefly, pathognomonic signs were: double wall for CE1 cysts; daughter cysts for CE2; detached parasitic membranes for CE3a; daughter cysts in a partially solid matrix including anechoic folded parasite membranes (“ball-of-wool” sign) for CE3b cysts; cysts completely filled by a solid matrix including anechoic folded parasite membranes for CE4 cysts, and egg-shell calcification of a CE4 cyst for the CE5 stage. Unilocular fluid-filled cysts with a visible single wall were classified as cystic lesions of uncertain etiology (referred to as CL in the WHO-IWGE classification) and excluded from the analyzed cohort. Only cysts with a clearly visible double wall sign were classified as CE1; only cysts showing a partial or complete egg-shell calcification of the wall together with pathognomonic “ball-of-wool” sign visible in the lesion were classified as CE5. Diagnosis and cyst stage classification were confirmed by experienced clinicians of the HERACLES project, by reviewing static images and videos. All patients had liver CE cysts, with the exception of two patients having peritoneal and one having muscular CE cysts. Cysts were classified as active if in stage CE1, CE2 and CE3b, and as inactive if in stage CE4 and CE5 [6]. Patients with CE3a cysts were not included in the cohort due to the impossibility to define by imaging the activity and viability of individual cysts in this stage [6]. If a patient was harbouring more than one cyst, the patient was classified as having active CE infection if the majority of cysts were in active stages; as having inactive CE if all cysts were in inactive stages, and excluded from the cohort if only a minority of the cysts were in active stages. No patient was receiving albendazole treatment at the time of sampling. In patients with active cysts who had received prior treatment with albendazole (n = 7/28), the last course was completed at least 2 years previously (median 4 years, range 2–22 years). In patients with inactive cysts who had received prior albendazole treatment (n = 12/43), the last administration was 3 years before or longer (median 7 years, range 3–15 years). As the majority of reactivations after albendazole treatment-induced inactivation occur within 2 years from inactivation, and that spontaneously-inactivated CE reactivates extremely rarely [37–39], the inactive CE group could be considered composed of stably inactive cysts. Control samples were from participants to the HERACLES screening campaigns with no lesions on US examination, who voluntarily donated their blood for research purposes. Importantly, these participants came from the same areas of CE cases, thus having the same background representation of possible concurrent infections.

Blood (10 ml) was obtained by venipuncture in K-EDTA containing tubes and stored at 4°C for a maximum of 6 hours before processing. To obtain plasma-DMSO samples (used for mass spectrometry), 5 ml blood was processed by sequential centrifugations at 600 x g, 1500 x g, and 10000 x g, each for 15 minutes at 4°C. DMSO was added at 5% v/v and samples were stored at -80°C until use. To obtain plasma samples (used for western blot validation), 5 ml of blood was processed by centrifugation at 1500 x g for 15’ at 4°C, and samples were stored at -80°C in 200 μl aliquots until use.

#### Exosome preparation

Analyses were performed on plasma pools in order to obtain sufficient volumes for EXO preparation, as well as to dilute out individual-specific EXO content not related to CE. We estimated that at least 20–25 individuals per pool were necessary to reach these goals. That is, a pool of more than 20 subjects is necessary to likely dilute out individual specificity, included undetected concurrent infections, letting common features related to CE infection emerge. Importantly, each CE pool was generated by mixing samples from several geographical origins of different countries (Romania, Turkey, Italy), and control pools were generated from CE-negative individuals from matching origins (i.e. CE-negative individuals found in the HERACLES screening campaigns). This made further unlikely that particular individual features and/or undetected infections would contribute in a significant proportion to each pool. Two pools of samples from patients with active CE cysts (23–25 samples and a total volume of 25–26 ml per each pool), three pools of samples from patients with inactive CE cysts (25–26 samples and 27–28 ml each pool), and two pools of samples from controls (respectively 25 ml and 50 ml each pool) were prepared. For Western Blot (WB) analysis, completely independent pools were generated from plasma samples collected at the centre for CE of San Matteo Hospital Foundation, Pavia, Italy. For the pool details used for proteomics and WB analyses refer to **Table 1**. The development of the protocol setup, analysing plasma pools from healthy people, is reported in the Supporting information files **(S1 and S2 Fig in S1 File, and S1 Table)**. Thus, the following protocol was used for EXO preparations. Frozen plasma samples were rapidly thawed at 37°C. All processes were then performed on ice and centrifugations at 4°C. After pooling, plasma was diluted 1:6 with filtered PBS and divided into SS34 tubes (Beckman Coulter, Inc.). MV separation was achieved by centrifuging 30 minutes at 20,000 x g. The supernatant was transferred into Ti70 tubes (Beckman Coulter, Inc.) and centrifuged 70 minutes at 100,000 x g. Then, the supernatant was discarded; pellets were washed with PBS, collected all in one tube and centrifuged again for 70 minutes at 100,000 x g. The EXO pellet was further transferred to TL100 tubes (Beckman Coulter, Inc.) and centrifuged again for 30 minutes at 100,000 × g. Finally, to better remove plasma contaminant proteins, the pellet was resuspended in 350 μl of PBS, mixed with 1.65 ml of 80% w/v sucrose, and loaded onto the bottom of a SW50.1 tube (Beckman Coulter, Inc.) for a total volume of 2 ml, 66% sucrose. Then, the sucrose gradient was stratified as follows: 0.9 ml of 50% sucrose, 0.6 ml of 30% sucrose, and 2 ml of 10% sucrose, and run at 210,000 x g for 18 h. Eleven fractions of 0.5 ml were collected and their refractive indices measured. Fractions with densities in the range (1.07–1.20 ± 0.02) g/ml (**S3 Fig in S1 File**), were further analysed by diluting 1:3 in Tris 10 mM pH 7.4 and centrifuged 50 minutes at 110,000 x g. The pellets were dissolved in 100 μl Tris 10mM pH 7.4, 0.1% SDS, vortexed, and Chlorophorm/Methanol extraction [40] was performed. Air-dried pellets were resuspended in 50 μl of 2x TCEP sample buffer [60 mM Tris-Cl pH 6.8, 2% SDS, 10% Glycerol, 50 mM (tris (2-carboxyethyl) phosphine), 0.01% Bromo Blue Phenol, and 0.1% Sodium Deoxycholate for 1h at 65°C.

#### Liquid Chromatography Tandem Mass Spectrometry

Protein identification and Protein Abundance Profiles (PAPs) were performed as already described [33] with some improvements. Briefly, 1/10 (5μl) of each sample was used for silver-staining on 4–12% Nu-PAGE gels (Invitrogen; Thermo Fisher Scientific, Inc.) to perform a quality control evaluating the protein mixture complexity and normalization (**S4 Fig in S1 File**); the remaining amount was resolved on 12% SDS-PAGE for 4 cm and the Coomassie stained lanes were divided into 8 bands. Trypsin was used at a concentration range of 4–10 ng/μl according to band intensity. Digestion was carried out in the presence of 0.1% sodium deoxycholate, removed from digests by acidification (1% trifluoroacetic acid), followed by ethyl acetate extraction [41]. Nano-RPLC was performed using a nano-HPLC 3000 Ultimate (Dionex; Thermo Fisher Scientific, Inc.) connected in line to LTQ-XL linear ion trap (Thermo Fisher Scientific, Inc.). Tryptic digests were first trapped on a C18 RP-precolumn (5 mm x 300 μm id; LC Packings-Dionex; Thermo Fisher Scientific, Inc.), and then run on a home-packed 15 cm x 75 μm id fused-silica column (8 PicoTip Emitter, New Objective) packed with Magic C18AQ (Michrom Bioresouces, Inc.) for chromatographic separation. Peptides were eluted at 0.3 μl/min along a 60 minutes’ linear gradient from 10% to 50% of buffer B (92% acetonitrile, 3% DMSO, 0.1% formic acid). Full-scan MS was set with a maximum injection time of 10 ms and 2 microscans. The five most intense ions were sequentially selected and fragmented in CID mode: maximum injection time of 100 ms; m/z 50–2000 mass range; minimum signal threshold of 200 counts; isolation width of 2; normalized collision energy of 35. Wideband was enabled and dynamic exclusion allowed a repeat count of 1 within 30 sec and exclusion time of 30 sec.

#### Data processing and Bioinformatics analysis

Spectra raw files (available as MassIve dataset at ftp://massive.ucsd.edu/MSV000085234) were analyzed by Sequest HT search engine with Proteome Discoverer 1.4 (Thermo Fisher Scientific, Inc.) against a homemade database constructed by joining the *Homo sapiens* SWISSprot (June 2016) and the *E*. *granulosus* Uniprot (released in June 2017) databases and also against the relative decoy database. The carboamidomethylation of cysteines was specified as fixed modification, the oxidation of methionine and phosphorylation of Serine, Threonine and Tyrosine were set as variable modifications; mass tolerance was set to 1 Da for precursor ion and 0.4 Da for fragment ions and a maximum of two missed cleavages was allowed. The Percolator tool was used for peptide validation based on the q-value and a high confidence was chosen, corresponding to a false discovery rate (FDR) ≤1% on peptide-level. Proteins were identified with a minimum of 1 unique peptide and at least 2 first-rank peptides. Keratins, serum albumin, complement factors, immunoglobulins, and fibrinogen were discarded as abundant contaminants. Mass spectra raw files of fractions with density (1.10–1.16 ± 0.02) g/ml were processed together to gain better identifications and quantifications of EXO proteins (fr 4, 5+6, 7 are respectively referred to as fr 5, 6, 7 later on). Protein relative abundance was assessed by label-free quantitative analysis based on Top3 method [42] and the abundance values in each fractions are reported in S2 Table. Only proteins quantified in at least two biological replicates were submitted to hierarchical average linkage clustering (Gene Cluster 3.0 [76]) using Pearson’s correlation (centered) as a measure of profile similarity. The dendrograms resulting from the analysis of samples from patients with active and inactive cysts were evaluated to select just those nodes carrying proteins with PAP Pearson Correlation values R ≥ 0.7 (p ≤ 0.05) for further analysis (**S6 Fig in S1 File**), and the specific PAP was defined as the median value of PAPs of replicas. In the case of control samples, all proteins identified in either one or both replicas were considered for further analysis without any filtering. Protein intensity PAP values were normalized by dividing each value over the highest value of the PAPs. Finally, protein sets from active/inactive CE and control samples were clustered together and the hierarchical dendrogram obtained was divided in 15 clusters on the basis of R coefficients.

The Principal Component Analysis (PCA) statistics and visualizations were carried out in R programing language [43], by means of the “prcomp” function using protein abundances as input of the analysed dataset.

The interaction networks were performed using STRING database Version 11.0 (string-db.com) set as follows: high confidence ≥ 0.7; only known interactions: curated database, experiments; and probability of protein-protein interaction (PPI) value < 10E-3. For STRING Enrichment analyses, if not differently reported, the PPI threshold value was set at 1.0E-16 and FDR < 1.0E-3. Network analyses were carried out using the String or Dynet applications installed in the open-source software-platform Cytoscape Version 3.7 [44]. As background, the entire human proteome was used, because a valid subproteome was not applicable. By using the entire human proteome as background and the filters just mentioned, a higher probability of false negative, but no false positive, enrichments could result.

#### Western Blot analysis

WB validation of protocol setup on healthy control pools were performed by using the following antibodies: anti-CD81 mouse monoclonal (sc-166029, Santa Cruz) 1:400 dilution; anti-Syntenin mouse monoclonal (sc-100336, Santa Cruz) 1:400 dilution and anti-TFR-1 mouse monoclonal (BD 612125, Biosciences) 1:1000 dilution; exposition time 1 min. The validation of MS analysis results and potential biomarkers were performed on plasma samples from active and inactive CE patients and control subjects collected in independent pools of about 30 ml (**Table 1**). As already described, these plasma samples, collected at the outpatient clinic for CE of San Matteo Hospital Foundation of Pavia in Italy, were not centrifuged at 10,000 x g before freezing and were stored in absence of DMSO. The following primary antibodies and concentrations were used: anti-Lyn mouse monoclonal (2796, Cell Signaling Tech.), 1:200 dilution; anti-Src mouse monoclonal (sc-8056 Santa Cruz), 1:200 dilution; anti-TFR-1 mouse monoclonal (BD 612125, Biosciences), 1:1000 dilution; and anti-Hsp70 (HSPA8) mouse monoclonal (sc-24, Santa Cruz), 1:500 dilution. Anti-mouse IgG (H+L) secondary antibody HRP conjugate (# 31430, Pierce) was used as secondary antibody. WB analysis was performed using MINI TRANS-BLOT Bio-Rad (Hercules, CA) apparatus at constant voltage (100V) for 1h, in transfer buffer (20% methanol, Tris 0,025M, Glycine0.192 M) onto Protran 0.22 microns membrane (Whatman, GE Healthcare Life Sciences). Primary and horseradish peroxidase-conjugated secondary antibody were incubated 1h in PBS-Tween (0.05%) 1% non-fat milk and membrane was developed using the ECL system (SuperSignalWest Pico, Thermo Fisher Scientific Inc. or Advansta Inc. K-12045-d20) according to manufacturer’s instructions.

## Results

### Exosome enrichments from control subjects and CE patient plasma pools

In this study we carried out a proteomic analysis of the exosomes circulating in the plasma of CE infected individuals, with either active (AP) or inactive CE (IP), and control subjects (Ctr), to identify potential biomarkers of CE infection and metacestode viability, and further to investigate the pathogen-host interplay communications. All CE patients screened in the HERACLES project survey [29, 30] were carefully characterized by US imaging and transitional cyst cases, patients under current or recent treatments, and patients with multiple cyst not classifiable in a clear-cut manner were excluded from the study. Finally, a total of 76 patients with inactive CE, 46 with active CE and 70 control subjects were enrolled. The study pipeline is reported in **Fig 1**, and described in the Materials and Methods section.

In spite of the great advances in proteomic sample preparation, the discovery of biomarkers in plasma still faces the challenge of the high dynamic range in protein concentrations. The analysis of circulating exosomes offers the advantage of a selected sub-proteome, essential to host-pathogen interplay, and free from very abundant plasma proteins. Nonetheless, the preparation of exosomes from plasma requires some shrewdness. As the isolation procedure significantly impacts EV yield from human plasma [45], we combined several protocol steps of Ultracentrifugation (UC) and Sucrose Density Gradient (DG) to obtain a preparation highly enriched in EXO. Further, the cargo and release amount of exosomes are highly sensitive to cellular status and environment [46, 47]. Thus, to overcome the inter-individual heterogeneity, the biomarker discovery process requires either the analysis of a very large number of individual samples or the adoption of a pool-strategy. We opted for the latter, and organized the subjects’ samples in pools of more than 20 individuals (**Table 1**). This experimental design not only affords sufficient starting volumes for exosome preparation, but also, and more importantly, provides a sufficient dilution of individual background differences (including any concurrent infections or concomitant disease eventually present in patient panels), which allows identifying “what is common”, in this case to CE infection, and more specifically to either active or inactive CE infection. This pool-strategy discovery was then combined with highly stringent criteria for filtering the protein datasets to be subjected to bioinformatics and further analyses, to identify potential biomarkers.

**Fig 2:**
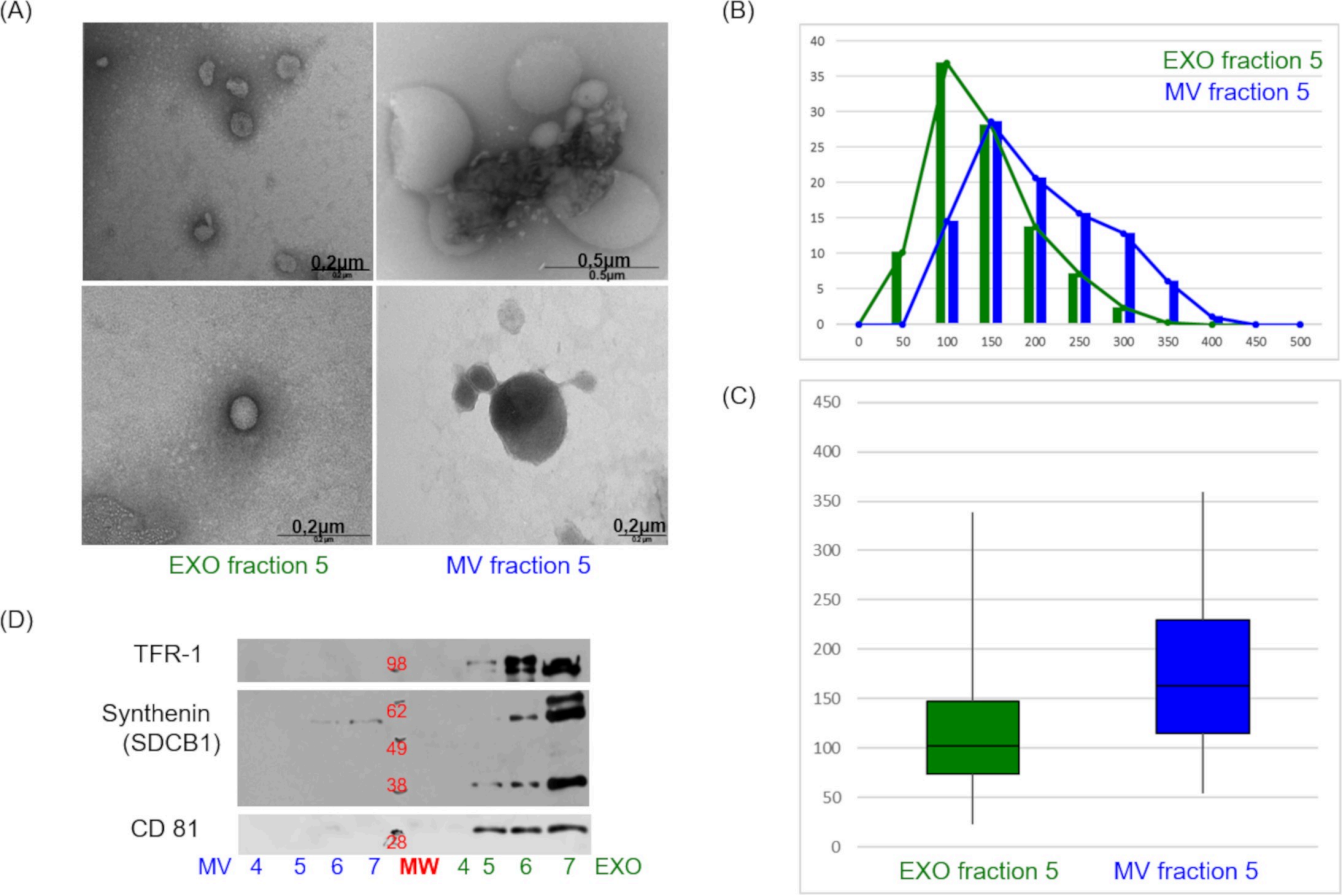
Exosome quality control. Biochemical characterization of EVs isolated from plasma pools from healthy subjects. (A) About 20% of sample volume from gradient fractions EXO/MV 5 was fixed for TEM analyses. (B) The diameter size distribution of particles is shown in percentage of total particles number and (C) in box-plot diagram. The median value of exosome diameter was 102 nm, while that of microvesicles was 158nm. (D) WB analysis of exosome markers CD81 (1:400), Synthenin (1:400), TFR 1 (1:1000), exposition time 1 min.

Firstly, the protocol for EXO preparation from human plasma was optimized on samples from healthy donors to obtain a proper separation of highly enriched EXO free from MV (**S1 File**). Of note, we inserted a UC step of 20,000 x g to remove MV before the standard UC at 100,000 x g to collect EXO. Both vesicle populations were analysed by mass spectrometry (MS), transmission electron microscopy (TEM) and western blot (WB). Representative TEM images are shown in **Fig 2A**. As expected the EXO diameter size distribution showed a sharper Gaussian curve than MV (**Fig 2B**) and the median value of EXO diameter resulted of 102 nm, while for MV was 158 nm (**Fig 2C**). The EXO markers TFR-1, Syntenin (SDBC1) and CD81 were detected in the EXO fractions but not in the MV fractions (**Fig 2D**), as expected. Further results obtained during the protocol setup are summarized in the Supporting Information (**S1 –S2 Fig in S1 File and MS data in S1 Table**).

Then, samples collected during the HERACLES project were processed [29, 30, 35, 48]. Each echinococcal cyst from each patient was carefully investigated by US and classified as described in the Materials and Methods to allow a coherent stratification for pool generation. Three pools of samples from patients with inactive cysts, two pools from patients with active cysts, and two pools from controls were analysed (**Table 1**). Density fractions in the exosome range of 1.07–1.20 g/ml (**S3 Fig in S1 File**) were analysed by mass spectrometry; proteins identified and quantified in all pooled samples are reported in **S2 Table**. A total of 322 proteins in active CE pools (AP) were identified and quantified in at least two replicate experiments; 240 in the inactive CE pools (IP), and only 107 in the control pools (Ctr). The AP samples yielded a higher number of identified proteins despite the smaller total volume (51 ml, divided into 2 pools) in comparison to IP samples (82 ml, divided into 3 pools) and Ctr samples (75 ml, divided into 2 pools). The number of identified proteins may be affected by several factors, such as the presence of highly abundant proteins, possible inconsistencies during the sample processing or peptide purification, or even instrument performance. However, taking into account the normalized presence of contaminant proteins in all of our samples (**S4 –S5 Fig in S1 File**) our results suggests a lower amount of circulating EXO in US-negative controls, as expected [15], but also a lower amount of protein associated with circulating EXO (either less EXO or lower protein cargo in EXO) in individuals with inactive CE cysts compared to patients with active CE cysts.

### Definition and general characterization of the protein datasets

**Fig 3:**
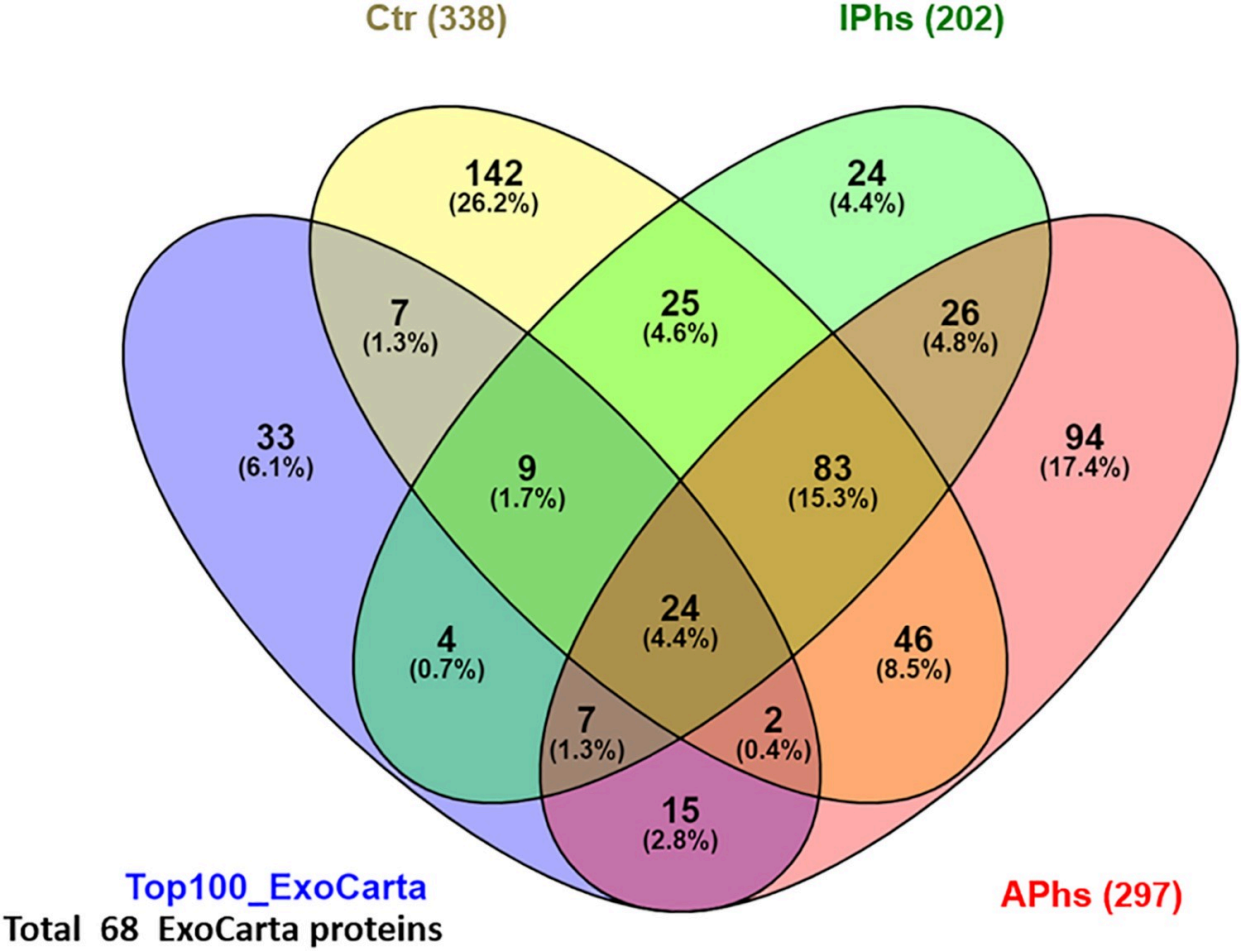
Venn diagram summarizing protein datasets results. APhs: selected human proteins from exosomes of patients with active CE; IPhs: selected human proteins from exosomes of patients with inactive CE; Ctr: control; Top100_ExoCarta: the 100 proteins most frequently identified in exosome proteomics studies.

Proteins identified in AP and/or IP were subjected to a stringent filtering step based on protein abundance profile (PAP, [33]) correlation clustering (**S6 Fig in S1 File and S2 Table**). Thus, for AP and IP, only those proteins that showed abundance profiles (across the density fractions) that were consistent (R ≥ 0.7) in at least two replicas were selected for further analysis. This process led to the selection and profiling of 302 proteins in AP (APs), 297 of human origin (APhs) and 5 of *E*. *granulosus* origin. In IP, 209 proteins were selected (IPs), 202 of human origin (IPhs) and 7 of *E*. *granulosus* origin. Proteins identified in Ctr pools (338, all of human origin) were not subjected to any filtering (even proteins identified in just one replica were included in the dataset) and were all considered for further analyses. The resulting subsets of human selected protein datasets (APhs, IPhs, Ctr) were then compared with the 100 genes most frequently identified in exosome proteomic studies and reported in the Top100 ExoCarta (www.exocarta.org [49]). A total of 68 proteins of the Top100 genes were identified in our analysis, confirming the good quality of the EXO enrichment (**Fig 3**). Then, a Principal Component Analysis (PCA) was performed in which datasets corresponding to the two replicas of each type of sample (APs, IPs and Ctr) were treated separately, in order to obtain a general view of the differences between them (**S7 Fig in S1 File and S3 Table**). As expected, the two APs replicas clustered closely together and separated well from the two IPs replicas, which also clustered together; in contrast, the two Ctr replicas were more widely spaced, reflecting the lack of filtering on them.

**Fig 4:**
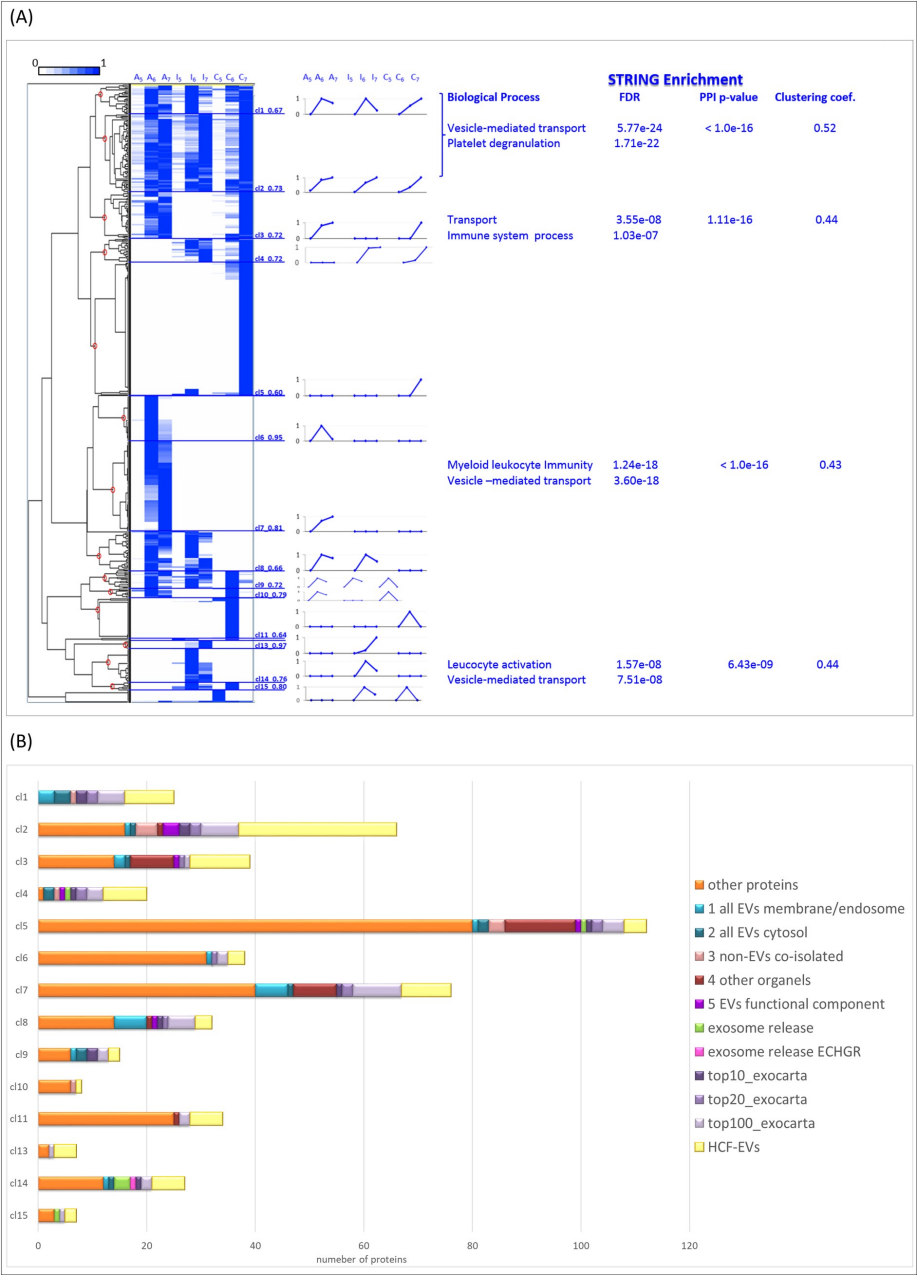
Complete clustering. **(A)** We clustered together the proteins in the APs and IPs datasets plus all the proteins identified in the two control replicas. From left to right: we divided the hierarchical dendrogram obtained (Cluster 3.0) in 15 clusters (circled in red in the dendrogram) (named cl1-cl15) by the values of the Pearson correlation coefficients which are reported next to each cluster number. The median normalized abundance profile of the proteins belonging to each cluster is shown for each sample (A_5-7_, I_5-7_, and C_5-7_ indicate active, inactive and control respectively, the subscripts 5–7 referring to fraction numbers). The String Enrichment analysis was performed for each cluster considering only: high-confidence interactions (> 0.7); known interactions: curated database, experiments; protein-protein interaction (PPI) probability and false Discovery rate (FDR) < 10E-3. Shared proteins identified in all samples (clusters cl1-cl2) resulted enriched in vesicle-mediated transport and platelet degranulation; proteins of cl3, identified in APs and Ctr only, were enriched in transport and immune system processes; the leukocyte-mediated immunity and vesicle-mediated transport pathways were enriched in clusters specific of APhs (cl6-cl7) and IPhs (cl13-14), with FDR values of 1E-18 and 1E-8 respectively. Control sample proteins fell in cl5 and cl11. *E*. *granulosus* proteins fell into the clusters corresponding to APs-selective (cl6-cl7) and IPs-selective proteins (cl13-cl14). **(B)** For each cluster, the number of proteins previously identified in the investigated literature are reported. The protein content-based EV characterization (numbered from 1 to 5 as reported in MISEV2018, [28]); the ones recognized to be involved in exosome release [52, 53], and we extended this definition also to the *E*. *granulosus* (ECHGR) Rab-11a; the proteins most frequently identified in exosome studies (top 10/20/100 genes from Exocarta [49]); the proteins already identified in CE-related EVs [54]; and finally, the remaining “other proteins”.

Then, APs, IPs and Ctr were organized together by clustering analysis. The resulting dendrogram showed an almost perfect grouping of common proteins identified in all sample types (clusters cl1-cl2), proteins specific to samples from CE-infected patients (cl8), specific to samples from patients with active CE (cl6-cl7), specific to inactive CE (cl13-cl14), and control-specific (cl5, cl11) (**Fig 4A and S4 Table**). Then, each cluster was analysed by String Enrichment in order to highlight Biological Processes. Considering only high confidence Known interactions with a probability PPI < 1E-3, we found significant enrichment of the pathways vesicle-mediated transport (FDR < 5.77E-24) and platelet degranulation (FDR < 1.71E-22) in the clusters of proteins common to all samples (cl1-cl2). In addition, significant enrichment of the pathways Transport (FDR < 3.55E-08) and Immune system processes (FDR < 1.03E-07) was detected in the cluster of APs and Ctr (cl3). Mainly leukocyte-mediated immunity and vesicle-mediated transport pathways were identified in clusters of APs (cl6-cl7) and IPs (cl13-14), with FDR values of 1E-18 and 1E-8 respectively. The lower significance found for IPs reflects in part the smaller number of specific proteins identified in IPs, and in part probably reflects a lower biological activity in this group than in APs.

Of note, all identified *E*. *granulosus* proteins (with the only exception of A0A068WS68, Protein fat free, identified only in IPs but found in the double-protein cluster 12) fell in the clusters corresponding to either APs or IPs proteins. The APs *E*. *granulosus* proteins (clusters cl6-cl7) were: A0A068WI65, Cation efflux protein zinc transporter; Q03341, Actin-2; A0A068WQT6, Expressed conserved protein; W6UMX7, Succinate dehydrogenase; A0A068WVR1, Tubulin alpha chain. The IPs-selective proteins (clusters cl13-cl14) were: A0A068WVB2, Probable tRNA N6-adenosine threonylcarbamoyltransferase; W6V5E3, Ubiquitin hydrolase FAF-X; U6JDZ7, Rab-11A; W6UFG1, ADP-ribosylation factor; W6UQH5, Tubulin beta chain; W6VD45, Phosphatidylinositol 4-kinase alpha. The annotation of *E*. *granulosus* proteins is still poor, but information recovered from the Uniprot (www.uniprot.org [50]) and the Wormbase Parasite (www.parasite.wormbase.org [51]) platforms show that all these proteins are compatible with EXO cargo and many were already identified in helminth EVs (**Table 2**).

**Table 2:** List of *E*. *granulosus* proteins identified. For each protein the presence in active (APs) or inactive (IPs) samples, the cluster annotation, the previous identification in EVs from HCF (HCF-EVs) and bibliographic references are reported.

Accession	Protein names	Gene	Organism	APs/IPs	Cluster	HCF-EVs	References
A0A068WI65	Cation efflux protein: zinc transporter	EgrG_001002800	ECHGR	APs	cl6_0.95		
A0A068WVR1	Tubulin alpha chain	EgrG_000042500	ECHGR	APs	cl7_0.81	x	52,59
Q03341	Actin-2	ACTII	ECHGR	APs	cl7_0.81	x	52,59
W6UMX7	Succinate dehydrogenase	EGR_02366	ECHGR	APs	cl7_0.81	x	6,52
A0A068WQT6	Expressed conserved protein	EgrG_000337300	ECHGR	APs	cl7_0.81		
A0A068WS68	Protein fat free	EgrG_000700220	ECHGR	IPs	cl12_0.69		
W6V5E3	Ubiquitin hydrolase FAF-X	EGR_03548	ECHGR	IPs	c13_0.97	x	52,59
A0A068WVB2	Probable tRNA N6-adenosine threonylcarbamoyltransferase	EgrG_000105300	ECHGR	IPs	c13_0.97		
U6JDZ7	Rab (Ras-related protein Rab-11A)	EGR_10509	ECHGR	IPs	cl14_0.76	x	52
W6UFG1	ADP-ribosylation factor	EGR_05324	ECHGR	IPs	cl14_0.76	x	52,6
W6UQH5	Tubulin beta chain	EGR_02075	ECHGR	IPs	cl14_0.76	x	52,59
W6VD45	Phosphatidylinositol 4-kinase alpha	EGR_00138	ECHGR	IPs	cl14_0.76		

Finally, we carried out a literature search (which did not attempt to be exhaustive) for previous identifications and annotations of the proteins in our final dataset, concerning exosome and/or CE-related EVs available proteomes. Besides the Top 10, 20 and 100 proteins reported in the Exocarta [49], we examined the last proposed EV characterization based on protein contents [28], the proteins involved in exosomes’ release [52, 53], and the proteins identified in previous studies on CE-related EVs [54].

This analysis showed that about 50% of the proteins of our final dataset were already identified/annotated, around 20% of them being specifically identified in CE-related EVs (**S8 Fig**). Single proteins’ identifications/annotations are reported in **S4 Table**, while the details for each cluster are summarized in **Fig 4B**. Importantly, residual contaminant proteins, such as abundant plasma lipoproteins or proteins associated with other intracellular compartments (corresponding to categories number 3 and 4 in the MISEV2018 guidelines [28]), amounted to 8% of the total proteins in our dataset, half of them falling respectively into clusters 2 and 5, which in turn includes proteins identified in all samples or only in the boundary fraction 7 of the control samples (**Fig 4A**). On the other hand, proteins previously identified in CE-related EVs were differently distributed in all clusters.

### The comparison of the CE patient and control subject datasets provides evidence that exosomes carry information on ongoing immune responses

**Fig 5:**
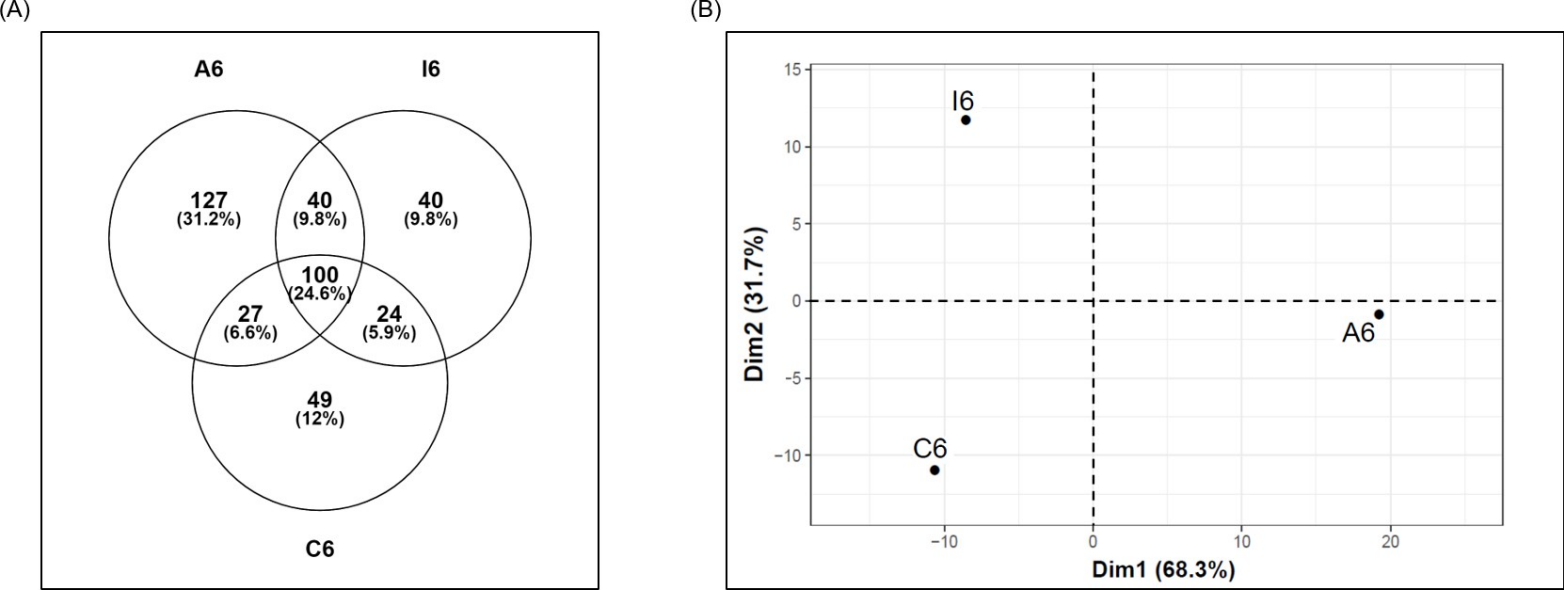
Comparative analysis of proteins detected in fraction 6 of Active, Inactive, and Control samples. **(A)** Venn diagram showing the distribution of the proteins detected in fractions 6 datasets. **(B)** Principal component analysis (PCA) plot of samples based on fraction 6 datasets, i.e. the median intensity values of the proteins identified in fractions 6 of the replicas of each sample. The position of conditions in the two generated main components is plotted. Variability explained by the first and second components of the PCA is indicated between brackets.

Besides the identification of *E*. *granulosus* proteins, we aimed to assess whether the results obtained on human proteins were yielding information relating to CE infection, as opposed to merely identifying proteins generally present in EXO. The side-by-side comparison between CE (APs and IPs) and Ctr faced the problem of higher probable level of contamination with non-exosome proteins in the Ctr (as observed in cl5 in Fig 4A and in C7 in S4 Fig). To circumvent this problem, the comparison was carried out taking into account only the middle density fractions (referred to as fraction 6, see Material and Methods), which are the most reliably enriched in EXO (density 1.10 ÷ 1.16 g/ml). Comparing the proteins identified in fraction 6 of the different datasets (407 proteins in total), we found that about 25% are shared among the three groups (100 proteins), 34% among CE samples (140 proteins), and about 30% are shared among active CE, or inactive CE, and controls. Whereas, the proteins identified specifically in APs, IPs and Ctr account respectively for about 30% (127 proteins), 10% (40 proteins) and 12% (49 proteins) (**Fig 5A**). It must be noted that since the criterion for including proteins in APs and IPs is more restrictive than the corresponding criterion for Ctr, any protein found in APs/IPs but not in Ctr might reflect characteristics of the infection.

Fraction 6 datasets, i.e. the median values of the APs/IPs/Ctr fraction 6 replicas, were subjected to PCA analysis with the purpose of obtaining information on the distances among the three biological situations. This analysis showed a clear separation of the three datasets, with a first dimension (accounting for the 68.3% of overall variation) distinguishing active CE, and a second dimension (accounting for the 31.7% of overall variation) differentiating inactive CE. However, no single protein contributed significantly to either dimension 1 or dimension 2; instead, many proteins, either dataset-specific or common to all datasets, shared the same contribution values (**Fig 5B and S5 Table_ Fraction 6_datasetsPCA**). These results suggested that the differences among the three datasets would not be ascribable to individual proteins, and could only partly be due to the presence of specific proteins in each dataset, instead they could mostly arise from the specific and complex interactions of the proteins in each dataset. Thus, we analysed the protein-protein interaction networks in the fractions 6 of APhs/IPhs/Ctr by STRING Enrichment, using the Cytoscape platform. This processing allowed the identification of enriched annotation terms, belonging to several categories (GO Biological Process, KEGG and Reactome Pathways), all based on high confidence, known protein-protein interaction (p < 1E-16 and FDR < 10E-3, using the entire human proteome as background). As expected, several terms confirmed the significant enrichment of patterns related to the ongoing immune response, but also indicated a significant difference between the CE samples, in line with the PCA result. Indeed, these included several terms which could explain the position of active CE along the PCA dimension 1 (Fig 5B), such as: the leukocyte mediated immunity (GO.0016192) and the immune effector process (GO.00002252), which were highly enriched in APhs compared with IPhs, and even more in comparison to Ctr; and the antigen processing and presentation of exogenous peptide antigen (GO.0019886) and the regulation of ERK1 and ERK2 cascade (GO.0070372) terms, which were only enriched in active samples. On the other hand, several terms could explain the position of inactive CE along the PCA dimension 2 (Fig 5B), such as: the wound healing term (GO.0042060), which was higher enriched in IPhs than in APhs, and even more in respect to Ctr; the interleukin-12-mediated signaling pathway (GO.0035722), more enriched in IPhs than in APhs, and not enriched in Ctr; and the regulation of TNF production (GO.0032680), the positive regulation of NF-kappaB transcription factor activity (GO.0051092) and the interferon-gamma-mediated signaling pathway (GO.0060333) terms, which were only enriched in inactive samples. The detailed FDR values and proteins involved in these terms are highlighted in **S5 Table_ Fraction6_Enrichment** where many other additional annotation terms, including immunologically relevant ones, are reported.

Thus, in addition to the presence of *E*. *granulosus* proteins, the human-origin proteins identified in exosomes from patients with CE provide unequivocal evidence of the ongoing immune response against the foreign agent, and also support a different response in active and inactive CE patients.

### The comparison of Active CE and Inactive CE protein networks suggests potential biomarkers of metacestode viability

Biomarkers allowing to discriminate active from inactive CE can conceivably include *E*. *granulosus* proteins and human proteins induced in these infection contexts. Interestingly, none of the *E*. *granulosus* proteins identified and selected were shared between IPs and APs datasets (**Table 2**), and thus all represent candidate markers of metacestode viability.

**Fig 6:**
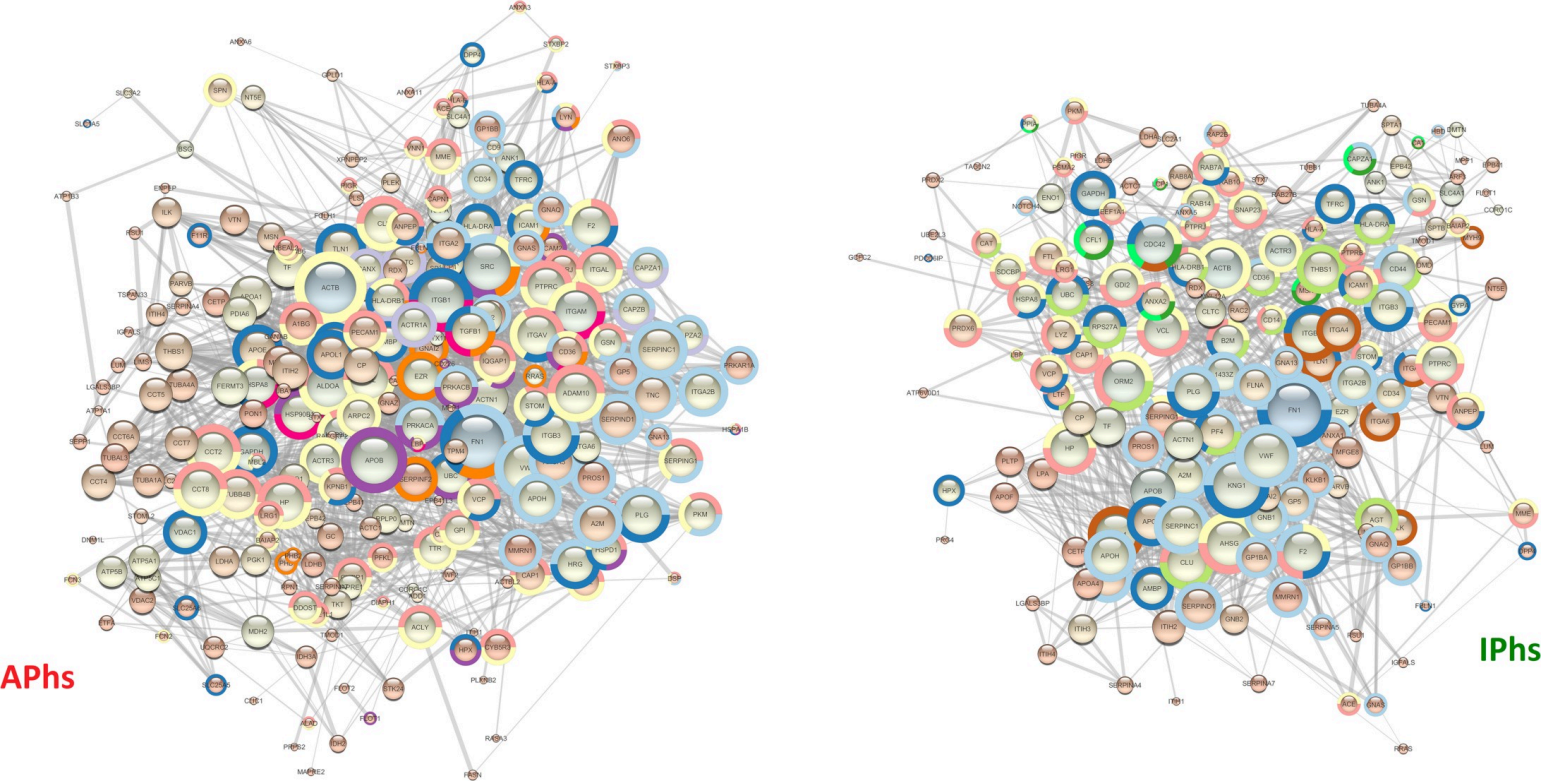
String Networks of APhs and IPhs. The String App on Cytoscape 3.1 platform was used to obtain the interaction networks of human proteins identified in the active (APhs) or inactive (IPhs) datasets respectively. STRING database Version 11.0 (string-db.com) was set as follows: high confidence ≥ 0.7; only known interaction: curated database; experiments. The node degree is visualized by **node size** and informs about the number of interactions of that protein; the betweenness centrality is visualized by **node colour,** with colours from orange to blue indicating increasing protein relevance in the network signalling; the edge score (confidence of protein-protein interaction) is visualised by direct proportional to the **edge size**. For STRING Enrichment analyses, the protein-protein interaction (PPI) probability threshold was set to 1.0E-16 and the FDR threshold to 1.0E10-3. Some of the enriched terms reported in Tab.2 are displayed by means of a colour surrounding the node, as follows. Specific APhs terms: Antigen processing and presentation of exogenous peptide antigen via MHC class II, **lilac;** Interleukin-4 and Interleukin-13 signalling, **fuchsia**; positive regulation of innate immune response, **purple;** regulation of ERK1 and ERK2 cascade, **orange.** Specific IPhs terms: gene and protein expression by JAK-STAT signalling after Interleukin-12 stimulation, **bright green;** Interferon-gamma-mediated signalling pathway, positive regulation of NF-kappaB transcription factor activity and regulation of TNF production, **light green**. Common terms: immune effector process, **yellow**; integrin-mediated signalling pathway, **brown;** interspecies interaction between organisms, **blue;** leukocyte mediated immunity, **pink;** wound healing: **sky blue**.

**Fig 7:**
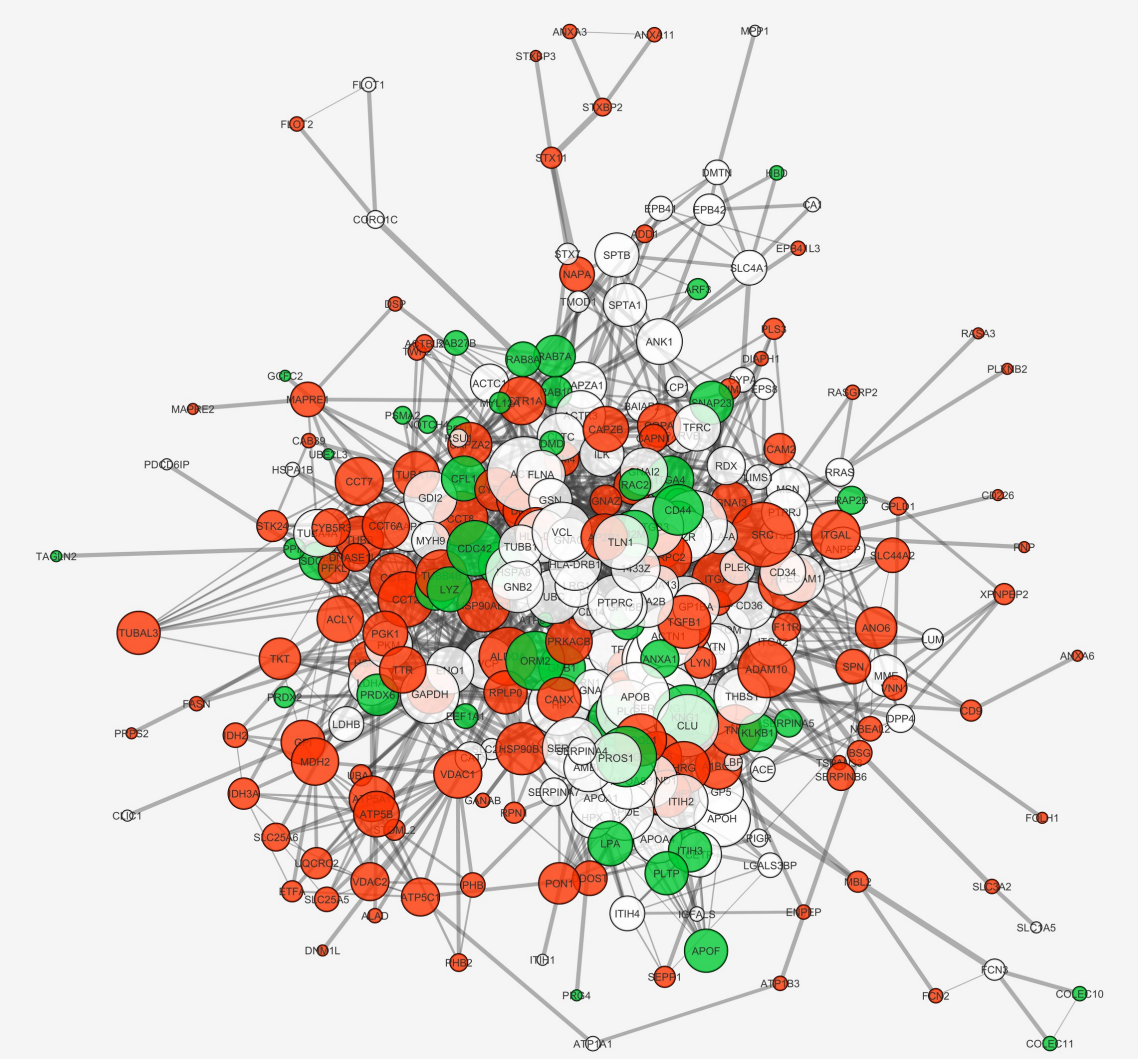
Dynet network. The APhs and IPhs networks were superimposed in one graph by the use of the Dynet application, to highlight patient group-specific and shared central proteins. Proteins identified only in samples from individuals with active CE are coloured in red, proteins identified only in inactive CE samples in green, and proteins identified in both samples in white. The node degree (number of interactions) is visualized by node size and the edge score (confidence of node interaction) is directly proportional to the edge size. On the right side are clearly visible the central hubs of APhs network, i.e. the proteins Src, TGF-β and the integrins α_M_, α_V,_ α_L_ (ITGAM, ITAV, ITAL).

Beside *E*. *granulosus* potential biomarkers, we aimed to identify potential biomarkers also among the selected human proteins, likely molecular actors of the different immunological responses in active and inactive CE. Therefore, we compared the protein-protein interaction STRING networks of APhs and IPhs in terms of node degree (**Fig 6**, node size), closeness and betweenness centrality (**Fig 6**, node colour from orange to blue), and edge score (**Fig 6**, edge size). No predicted interactions were considered, but only the high confidence known ones, based on experimental data and curated databases. The comparison of the two networks made it evident that the differences were not merely attributable either to the amount or to the specificity of the identified proteins in the two datasets. Rather, differences arose from the presence of specific proteins and central hubs in the respective networks APhs or IPhs. For APhs, these included the SFKs, Src and Lyn, the immune-modulatory cytokine TGF-β1, and the integrins α_M_, α_V,_ α_L_ (ITGAM, ITAV, ITAL). For IPhs these included mainly the small GTPase Cdc42. In order to compare finely the two networks, we used the Dynet Network analysis, which allows the production of a unique graph by overlying both APhs and IPhs networks (**Fig 7**). The great density and the relative short radius of the graph, together with the shared (in white) proteins at the centre, confirmed the overlap of the majority of connected patterns. However, several specific proteins either for APhs (in red) either for IPhs (in green) were clearly recognizable as the specific hub proteins (bigger circles) just mentioned, showing high degree and centrality parameters (networks details are summarized in **S6 Table**).

**Fig 8:**
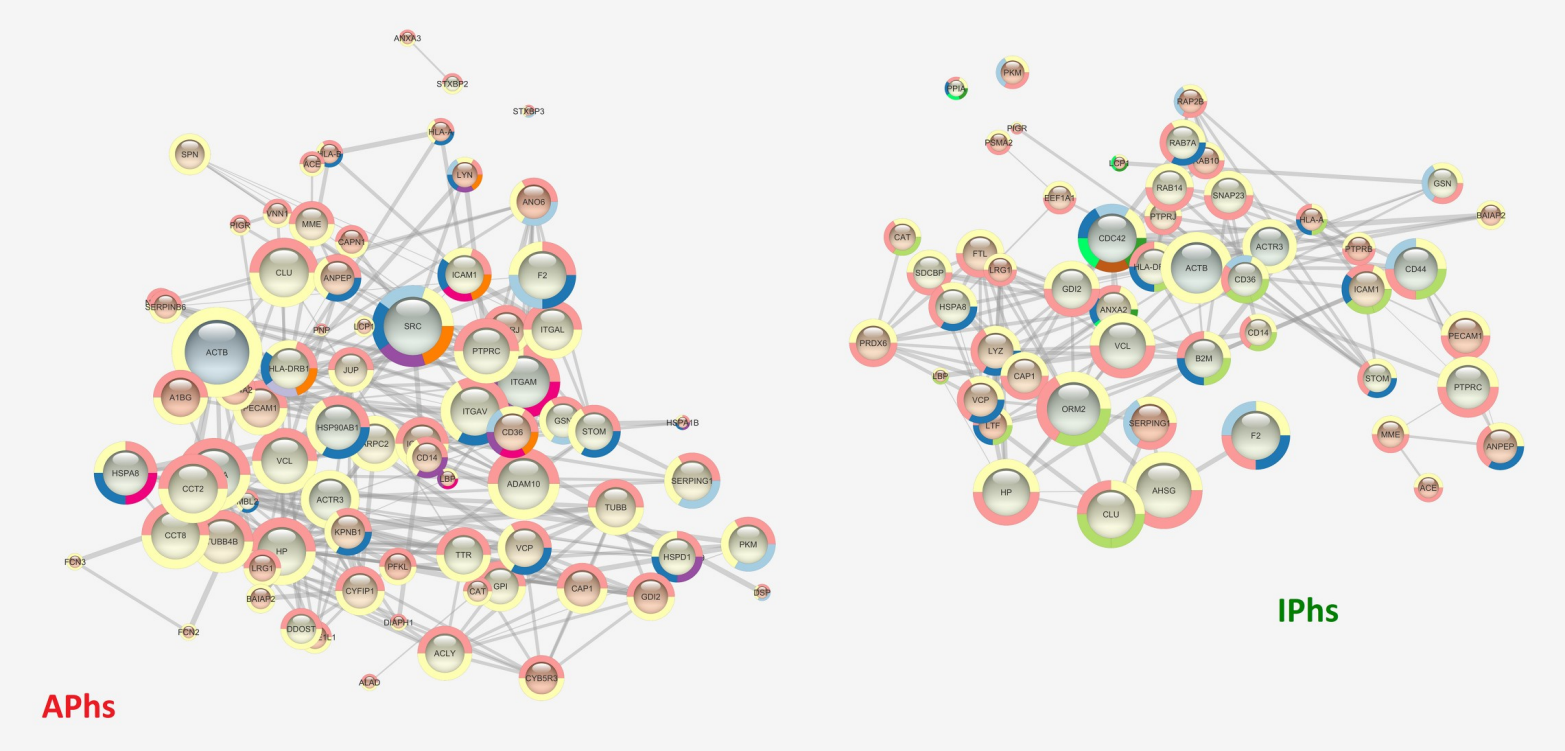
Immune effector networks. The STRING networks corresponding to the GO Term “Immune effector” for APhs (81 proteins; FDR = 9.45E-35) and IPhs (55 proteins; FDR = 7.62E-24) are shown. Besides the different number of proteins involved in the two networks, the term results highly enriched in both datasets but the signalling is based on different proteins: Src, Lyn and certain Integrins (ITGAV, ITGAM, ITGAL) in APhs *vs* Cdc-42, several Rab proteins and Annexin 2 in IPhs.

**Fig 9:**
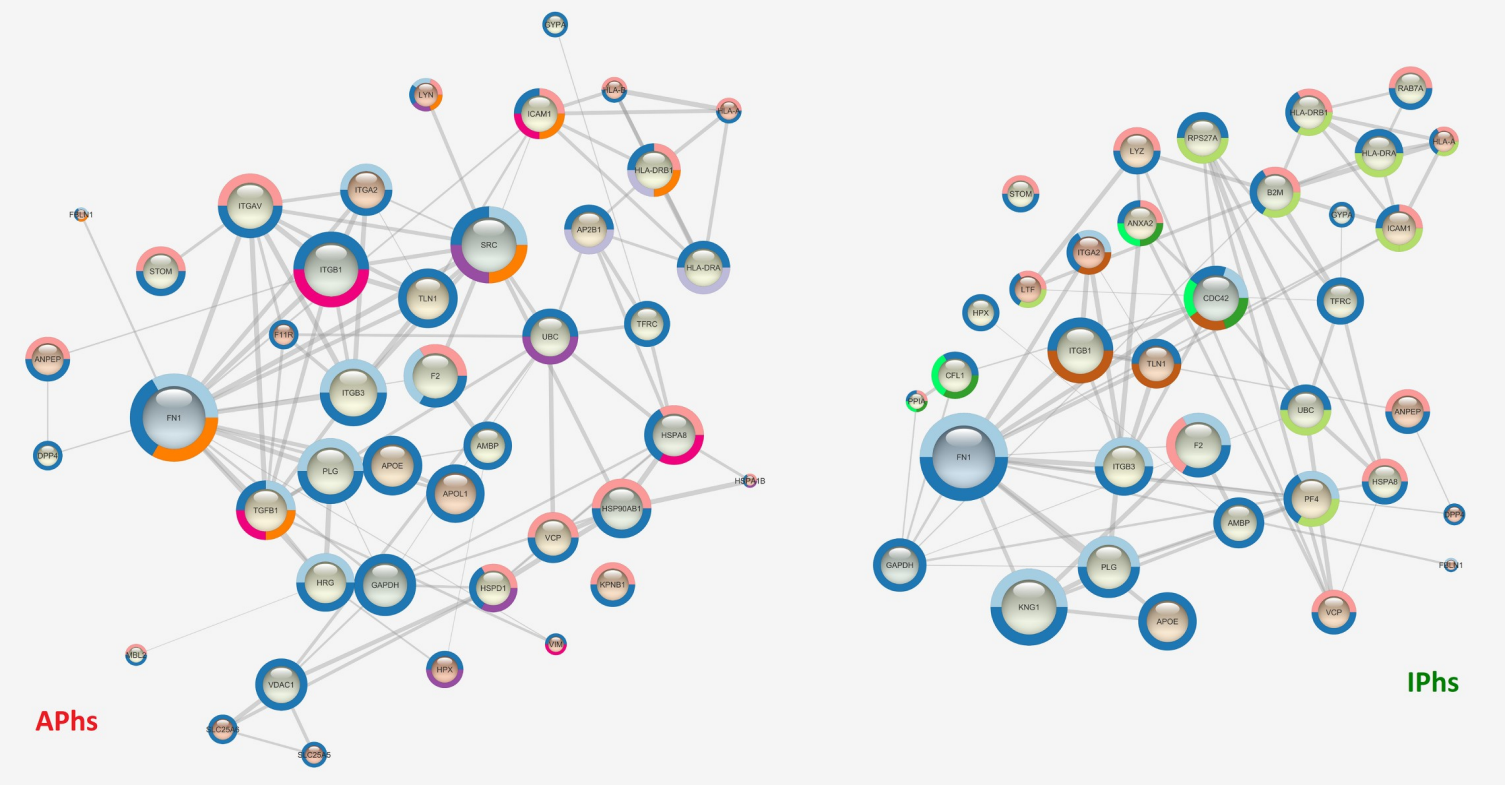
Networks of interspecies interaction between organisms. The networks of GO Biological Process Term “Interspecies interaction between organisms” for APhs (46 proteins; FDR = 4.37E-14) and IPhs (39 proteins; FDR = 2.97E-15) are shown. The two networks are very similar in the protein number and interaction confidence, but the signalling is based on different proteins: Src, Lyn, several Integrins (ITGAV, ITGB1, ITGB3, ITGA2), TGF-β1 in APhs *vs* Ccd-42, Annexin 2 in IPhs.

We next analysed and compared the APhs/IPhs Networks by STRING Enrichments, using the Cytoscape platform and the threshold already mentioned. This processing included the identification of GO Biological Process or Reactome Pathway terms significantly enriched in APhs or IPhs, i.e. identified either only in one dataset or in both, but with a difference of at least 10E-3 in the FDR value. This comparison confirmed the trends already observed in the network enrichment analysis of the fractions 6 datasets, and further suggested a more intense cellular activity in APhs related to energy production, cell communication, vesicle-mediated transport, biogenesis, metabolism, assembly, and localization (**S7 Table**). Interestingly, several pathways related to the myeloid leukocyte immune response were differentially enriched in APhs compared to IPhs. These included TLR signalling pathway, neutrophil-mediated immunity, Fc-gamma receptor signalling pathway involved in phagocytosis, antigen processing and presentation of exogenous peptides via MHC class II, integrin-mediated signalling pathway, and finally, the regulation of MAPK/ERK1-2 cascade, which is characterized by the presence of the SFKs Src and Lyn, and the TGF-β1 cytokine. Detecting statistically significant enrichments in IPhs could be expected to be more difficult than in APhs, given the smaller dataset size. However, several GO Biological Process were found to be selectively enriched in IPhs, including wound healing, interaction with symbionts, regulation of nitrogen compound metabolic process, and maintenance of cell polarity. Of specific immunological interest, IPhs were differentially enriched in processes related to the expression and/or response to the cytokines interleukin (IL)-12, interferon (IFN)-γ, tumor necrosis factor (TNF)-α, and the positive regulation of NF-κB pathway. Similar results were obtained analysing the Reactome Pathways where, of note, we found the enrichment of IL-4 and IL-13 signalling in APhs whereas the Gene and protein expression by JAK-STAT signalling after IL-12 stimulation was more enriched in the IPhs (these results are summarized in **Table 3)**. Thus, in general, the enrichment analysis confirmed *in vivo* the conclusions or trends already observed in conventional analyses of immune responses in CE patients [8, 55–60] suggesting a strong myeloid cell response in a Th1/Th2 immune environment in active CE, whereas inactive CE could be associated with a wound healing response in a Th1/inflammatory environment. Then, focusing on the specific hub proteins and their relative networks, we found several processes that were differently organized in active and inactive CE, i.e. centred on Src tyrosine kinase in the case of APhs and on Cdc42 in the case of IPhs. This is evident, for example, either in the Immune effector response term (GO.0002252, **Fig 8**), which resulted more enriched in active CE (highlighted in yellow in **Table 3**), and in the Interspecies interaction between organisms term (GO.0044419, **Fig 9**), which instead resulted equally enriched in the two datasets (highlighted in blue in **Table 3**).

**Table 3:** A selection of GO Biological Process and Reactome Pathways enriched in APhs and/or IPhs. For each Process or Pathway listed, the number of genes belonging to the Term and the false discovery rate (FDR) probability are reported. The colours used in Fig 6 are used to highlight the specific GO terms and are reported in the last column.

Term name	Description	APhs_# genes	APhs_FDR	IPhs_# genes	IPhs_FDR	Color in Fig 3
GO.0030036	actin cytoskeleton organization	36	1,61E-14	30	1,46E-14	
GO.0032956	regulation of actin cytoskeleton organization	25	5,65E-10	18	7,57E-08	
GO.0038096	antigen processing and presentation of exogenous peptide antigen via MHC class II	10	4,43E-05	-	-	lilac
GO.0007166	cell surface receptor signaling pathway	75	4,84E-10	57	7,66E-10	
GO.0009611	cellular component organization or biogenesis	156	3,64E-18	-	-	
GO.0019886	cytokine production	10	5,50E-04	-	-	
GO.0019221	cytokine-mediated signaling pathway	27	4,34E-05	26	1,21E-07	
GO.0007163	establishment or maintenance of cell polarity	11	4,80E-04	13	5,47E-07	
GO.0006909	Fc-gamma receptor signaling pathway involved in phagocytosis	9	3,91E-05	-	-	
HSA-8950505	Gene and protein expression by JAK-STAT signaling after Interleukin-12 stimulation			8	5,07E-07	bright green
GO.0002252	immune effector process	81	9,45E-35	56	1,10E-24	yellow
GO.0007229	integrin-mediated signaling pathway	14	5,42E-09	10	7,16E-07	brown
GO.0051702	interaction with symbiont	9	4,68E-05	12	2,11E-09	
GO.0060333	interferon-gamma-mediated signaling pathway	-	-	6	9,10E-04	light green
GO.0035722	interleukin-12-mediated signaling pathway	6	9,20E-04	8	1,36E-06	
HSA-6785807	Interleukin-4 and Interleukin-13 signaling	9	4,50E-04	-	-	fuchsia
GO.0044419	interspecies interaction between organisms	46	4,37E-14	39	2,97E-15	blue
GO.0002443	leukocyte mediated immunity	71	2,47E-36	47	7,12E-24	pink
GO.0051851	modification by host of symbiont morphology or physiology	7	1,10E-03	11	1,57E-08	
GO.0010951	negative regulation of endopeptidase activity	22	2,26E-09	24	2,29E-14	
GO.0051172	negative regulation of nitrogen compound metabolic process	58	5,90E-04	52	5,51E-07	
GO.0002446	neutrophil mediated immunity	63	1,28E-34	43	5,50E-24	
GO.0071840	phagocytosis	29	1,25E-17	17	7,66E-10	
GO.0045089	positive regulation of innate immune response	19	6,60E-07	-	-	purple
GO.0051092	positive regulation of NF-kappaB transcription factor activity	-	-	9	2,30E-04	light green
GO.0002224	regulation of ERK1 and ERK2 cascade	14	8,00E-04	-	-	orange
GO.0032680	regulation of TNF production	-	-	9	5,71E-05	light green
GO.0009605	response to external stimulus	62	6,39E-08	54	5,10E-11	
GO.0042060	response to wounding	47	4,33E-19	42	9,15E-22	
GO.0001816	toll-like receptor signaling pathway	9	1,20E-04	-	-	
GO.0016192	vesicle-mediated transport	140	1,33E-62	102	1,60E-49	
GO.0070372	wound healing	45	3,69E-20	41	2,78E-23	sky blue

**Fig 10:**
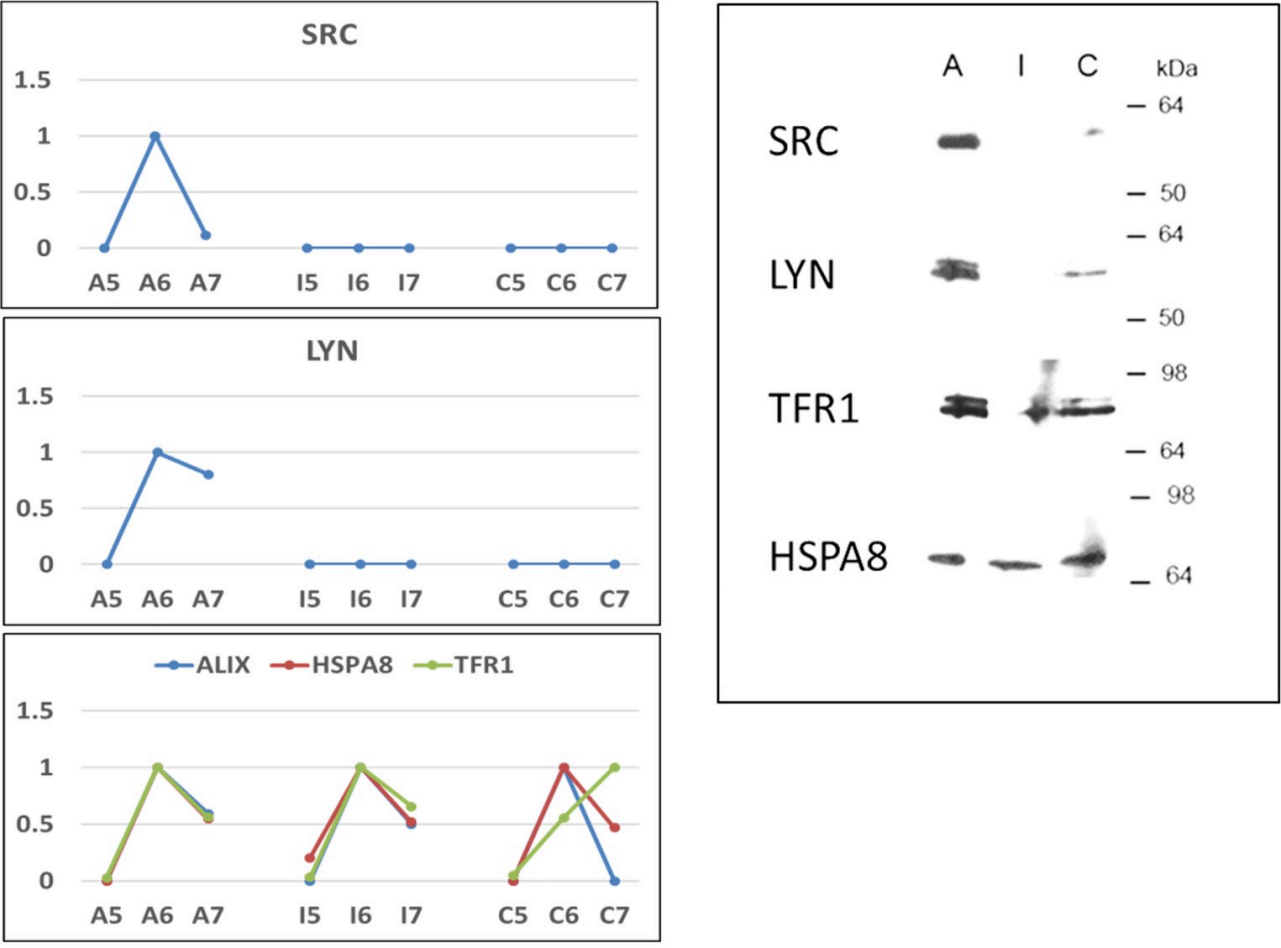
Validation by WB of some exosome markers. We validated the presence of SFK Lyn and Src specifically in active CE samples on a completely independent EXO preparation. On the left are reported the PAPs of EXO markers (Alix, Tfr1, HspA8) and SFK Lyn and Src identified by proteomics as reported in S4 Table. On the right, the WB performed on fraction 6 of new EXO preparation performed from pools of plasma samples (Tab.1) collected at the outpatient clinic for CE of San Matteo Hospital Foundation (Pavia, Italy). These plasma samples were not processed before freezing (not centrifuged at 10,000 x g), and were stored in absence of DMSO. We were able to confirm the presence of EXO markers such as Tfr-1 and HspA8 in all samples, while the presence of Src and Lyn was detected in samples from patients with active CE, at much lower levels in controls, but not in samples from patients with inactive CE.

Finally, we selected the SFKs Src and Lyn as promising marker candidates of active CE, and probed their specific presence in samples from a completely independent EXO preparation, i.e. from plasma samples processed and stored differently from the ones used in the proteomic discovery phase (ref. Materials and Methods section). We detected the presence of Src and Lyn in samples from active CE patients, but not in samples from inactive CE patients, while much weaker signals were detectable in controls. The presence of the EXO markers Tfr1 and Hsc70 (HSPA8) in all samples was also confirmed (**Fig 10**). The faint signal of the potential biomarkers Src and Lyn in the control could be attributed to a higher sensitivity of the antibodies used in WB, although extra-abdominal CE infection (i.e. not identifiable by US) in individuals forming the control group cannot be completely excluded.

## Discussion

In this study, we carried out a proteomic analysis of the exosomes circulating in the plasma of active/inactive CE infected individuals and control samples. The pool strategy combined with the protocol setup and the PAP approach allowed to select the most reliable exosome protein identifications resulting in the analysis of a robust dataset. The results individuated potential biomarkers of CE infection and more specifically of metacestode viability either among the *E*. *granulosus* identified proteins or among human host proteins. These candidates, once validated on a larger cohort, would be a valuable improvement for CE diagnosis and management. In addition, our results provide further information for the understanding of immune regulation elicited by the larval stage of *E*. *granulosus* and shuttled by exosomes.

Among the 11 *E*. *granulosus* identified proteins (**Table 2**), Actins, Tubulins and Ubiquitins have been already reported as proteins commonly identified in exosome-like vesicles, released by other helminths or in proteomic studies of hydatid cyst fluids [54, 61]. Rab-11A was previously identified in extracellular vesicles derived from hydatid cyst fluid (HCF-EVs) and in extracellular vesicles derived from protoscolex culture supernatant (PCS-EVs) [54]. ADP-ribosylation factor was previously identified in HCF-EVs and in PCS-EVs [54, 62] (all detailed information are summarized in **Table 2**). Contrasting results, concerning proteins previously identified in PCS-EVs [54] and here reported as specific of inactive CE, can be explained by several reasons: a) the higher number of analysed samples; b) the filters applied to our datasets, in particular to many proteins identified just in the first replica of active CE; c) the potential differences between *in vitro* cultures and *in vivo* conditions.

Among the proteins associated with active CE, two are of particular interest. The first is the conserved protein A0A068WQT6, because is characterized by a protein sequence very specific to the parasite, with no detectable homologues outside cestodes. The other is the succinate dehydrogenase enzyme (W6UMX7), which has been already identified in HCF-EVs, and deserves a special comment because both its substrate and product (i.e. respectively succinate and fumarate), were already described as possible metabolite markers of *E*. *granulosus* active cysts [6, 54].

Among the *E*. *granulosus* identified proteins associated with inactive CE, the Rab-11A (U6JDZ7) and ADP-ribosylation factor (W6UFG1) were identified in the same cluster (cl14) showing very similar PAPs and are both involved in the endocytosis and vesicles trafficking pathways, supporting the idea of a vesicles-cargo exchange between host and parasite cells.

Monoclonal antibodies against these interesting proteins need to be produced and further studies are needed to validate these *E*. *granulosus* proteins identified as possible biomarkers of CE infection and metacestode viability.

In comparison to our study, all previous works had identified proteins in extracellular vesicles released by protoscoleces *in vitro* or present in hydatid cyst fluid, and always considered few individual cases or animal models. As far we know, this is the first study conducted on a large, well-characterized cohort, which confirms that some of those proteins indeed circulate in the human host blood *in vivo*. We cannot exclude the presence, in our pools, of parasite-derived vesicles, but the low number and type of *E*. *granulosus* proteins identified likely suggest that we revealed the presence of these proteins within human-derived exosomes. The finding of parasite proteins in human circulating exosomes suggests that parasite exosomes could cross the laminated layer (reviewed in [63, 64]) *in vivo*, as already suggested [65]. These proteins might be safely shuttled, preventing degradation, by parasite-exosomes from parasite cells to host cells, and by host-exosomes among host cells.

The analysis of human proteins in exosomes from CE patients, based on the Network analysis and Biological Process and Reactome Pathway enrichments, suggested that active CE is associated with a stronger myeloid cell response and ERK1/2 regulated response, in a Th1/Th2 immune environment with an evident regulatory component. In contrast, inactive CE would be associated with a wound healing response in a Th1/inflammatory environment (**Table 3**). As already mentioned, these results obtained by the analysis of *in vivo* circulating exosome cargos are consistent with previously reported data obtained by *in vitro* cultures or serology, confirming a Th2 response correlated with disease susceptibility and Th1/Th2 coexistence in active cysts, whereas a Th1 response correlated with protective immunity in the presence of inactive cysts [8, 59]. In particular, it has been reported that *E*. *granulosus*-specific T cell clones, derived from active CE patients, produced both the Th1 cytokine IFN-gamma and the Th2 cytokine IL-4, whereas those derived from patients with inactive CE produced only IFN-gamma [56]. Additionally, stronger IL-4 responses in patients with active compared to inactive CE have been observed at protein level in serum and supernatants of ex-vivo antigen-stimulated blood cells, and at mRNA level in peripheral blood mononuclear cells [58, 60]. Further, a trend towards higher frequencies of CD4+ T cells expressing Th2 cytokines (IL-4/IL-5/IL-13) in patients with active compared to inactive CE was also observed by Petrone et al [55]. Interestingly, in this last study a trend was also observed towards higher frequencies of the inflammatory cytokine TNF-alpha in patients with inactive compared to active CE.

The contrasting immune response profiles suggested by the networks analysis helped interpret the result obtained in the PCA comparison of samples (**Fig 5B**). Indeed, the separation of active CE, away from both inactive CE and control, along dimension 1 (almost 70% of total variation) could be mainly attributed to the Th2 environment and the regulatory component; whereas the separation of inactive CE, away from active CE, and even more from controls, along dimension 2 (almost 30% of the total variability), could be ascribed to the inflammatory/wound healing environment. The association between viable parasites and partial control of inflammation, and on the other hand, between the presence of degenerating parasites and strong inflammation, is a known trend in larval cestode infections [66]. This evaluation can be further strengthened by focusing on some individual proteins of particular interest, which form part of pathways distinctively enriched in active vs inactive CE samples.

In agreement with a stronger regulatory element in active CE, proteins found in APhs but not IPhs datasets include the central immune-suppressive cytokine TGF-β, which stimulates the differentiation of naive T cells into FoxP3^+^ regulatory T and regulatory B cells, inducing immune balance and tolerance [67, 68]. Increased local or systemic TGF-β expression has been repeatedly reported in human and experimental CE, and associated with negative regulation of responses to the parasite [66, 69–72]. TGF-β is secreted in latent form, which must be post-translationally activated with the participation of α_v_ integrins. The α_v_ integrin subunit is another protein present in APhs but not IPhs and central in the active CE network (**Fig 7**). In exosomes, it is known to be involved in the preparation of a pre-metastatic niche in liver by cancer cells with hepatic tropism [73]. Thus, the selective finding of α_v_ in APhs is also of interest in the context of the observation that *E*. *granulosus* larvae localized in the peritoneal cavity of experimentally infected mice predispose for metastasis to the liver of subcutaneously implanted breast cancer cells [74]. Another protein selectively found in APhs was basigin (CD147). High expression of this plasma membrane protein marks an activated, strongly suppressive subset of FoxP3^+^ regulatory T cells [75, 76]. Also, basigin carried in exosomes has been shown to promote angiogenesis [77].

In addition, we found several proteins and statistically enriched processes suggesting a sustained activation of neutrophil mediated immunity and a stronger innate immune response [78] in samples from patients with active CE than in those with inactive CE (**Table 2**). Migration of monocytes and other immune cells into tissues occurs in response to gradients of chemokines that stimulate chemotaxis by activating GPCRs and downstream signalling pathways. Several studies support the evidence that inhibition of myeloid cell migration by IFN-γ *in vivo* serves to restrain infiltration of inflamed tissues and control associated tissue damage, thereby attenuating the severity of disease models of human autoimmune diseases. To exert this important negative control of homeostasis and chemokine-induced monocyte migration, IFN-γ antagonizes TGF-β and decreases the remodelling of the actin cytoskeleton brought about by signalling via GPCRs, the Rho-family GTPases Rac2/Cdc-42, and JAK-STAT [79]. In particular, IFN-γ fixes Rac2 and Cdc42 in an active state, thereby preventing the cycling between active and inactive forms that is required for dynamic actin remodelling and migration [80, 81]. In this context, the identification of TGF-β specifically in exosomes from active CE samples and, in contrast, the finding of proteins related to the IFN-γ and JAK-STAT signalling pathways, as well as Rac2 and Cdc-42, as specific features of inactive CE samples, suggests that chemotaxis and myeloid cell migration may be differentially regulated in the two situations, as supported by the enrichment of the regulation of actin cytoskeleton organization term (GO.0032956, Table 3).

Possibly consistent with the stronger inflammatory activity in inactive CE is also the identification of Annexin A2 as a central protein in IPhs networks (Figs 6–9). This cell cortex protein, often found in association with extracellular vesicles [82, 83], was previously found to be present in high abundance at the host-parasite interface in natural bovine CE [84]. There, the parasite usually displays little viability and fertility [66], suggesting a biological context similar to inactive human CE.

Finally, we identified exclusively in APhs the SFKs, Src and Lyn. SFKs are implicated in numerous receptor-mediated signalling pathways, including T and B cell receptors and neutrophil G protein-coupled receptor (GPCR). Lyn is thought to act as a master negative controller of activating signaling via immunoreceptors, through its activation of the inhibiting phosphatase SHP-1 [85, 86]. Accordingly, dysregulation of Lyn in mice results in myeloproliferation and autoimmunity [87–91]. It has been reported that Lyn exerts regulatory control on Th2 differentiation although it does not seem directly involved in the inhibition of Ras-Erk MAPK signalling [92]. In the field of helminth immune-evasion, Lyn/SHP-1 are known to be involved in the negative regulation of B Cell Receptor and inhibition of the MAPK/ERK signal pathway which result in the subversion of the immunological response from Th1 to Th2 induced by the secreted filarial protein ES-62 [93]. In this study we found several processes, specifically enriched in APhs or differently organized in active than inactive CE, which were centred on Src tyrosine kinase. Thus, using new independent pools, we tested the SFKs Src and Lyn as potential plasma biomarkers for CE and particularly for the metacestode viability, and obtained the encouraging result of validating their presence only in active CE.

In summary, our results provide *in vivo* evidence that supports and integrates the previous reported different response patterns in active *vs* inactive CE. Active CE would be associated with significant immune activity and in particular myeloid cell activity, in a combined Th1-Th2 environment with a strong regulatory component; some specific features of this scenario appear to be a strong antigen-presenting cell activity, activation of the ERK1/2 pathway, and the TGF-β/FoxP3^+^ regulatory T cell axis. This scenario suggests that the immune evasion in CE is a very active process (the parasite elicits strong responses but these are efficiently controlled), and not a weak response intrinsically associated to the physical sequestration of the parasite. In contrast, inactive CE appears to be associated with less strong antigen-presenting cell activity, stronger control of chemotaxis, in a Th1 and inflammatory environment with elements of wound healing responses. Importantly, we suggested some potential biomarkers of *E*. *granulosus* origin as well as some human proteins, differently shuttled in active and inactive exosomes, which appear central hubs in organizing and carrying different immunological messages, and in particular, we confirmed the SFKs Src and Lyn as potential markers of active CE in independent pools.

Our results will foster future studies to advance the understanding of the complex and still largely unknown aspects of the sophisticated host-parasite interplay during CE infection, and suggest new molecular candidates for CE diagnosis and therapy. Further studies are necessary to test the potential biomarkers here identified and their interaction with the immune-tolerance mechanisms suggested by our results.

## Conclusion

To our knowledge, this is the first proteomic study investigating the *in vivo* human immune response to CE infection conveyed by the peripheral blood circulating exosomes from a large cohort of clinically well-characterized samples [6, 38, 39, 94]. We preliminarily identified some *E*. *granulosus* and human proteins as potential CE and metacestode viability markers. These include, for active CE, the *E*. *granulosus* proteins Succinate dehydrogenase (W6UMX7) and A0A068WQT6 (Expressed conserved protein), and the human SFKs Src and Lyn. Further studies are needed to validate these candidates. In terms of immunological information, the study suggests the presence of a Th2 and Treg-oriented immunological profile in active CE in contract with a Th1/inflammatory/wound healing profile in inactive CE. Thus, we confirmed the complex immunoregulatory response to *E*. *granulosus*, and the important role of exosomes in this finely tuned immune-modulation, which allows for a long-lasting and asymptomatic coexistence. Such a masterful adaptation is the result of the long coevolution of helminths within their hosts, sustaining a historical dialogue of millions of years, at least partly conveyed through extracellular vesicles.

## Supporting information

S1 File Supporting information and S1-S8 Figures. Supplementary information, figures and tables referred to exosome preparation and quality control of samples.
(DOCX)

S1 Table Table protocol setup. Quantitative proteomic profiles of plasma microvesicles (MV) and exosomes (EXO) from healthy samples analysed to setup the exosome preparation protocol.
(XLSX)

S2 Table Proteins identified active-inactive-controls processing. The file contains three sheets, respectively named “active”, “inactive”, “controls”, in which all the identified proteins in each sample pool are listed and the relative processing to define the selected datasets for each sample are reported. In the fourth sheet the table used for the final cluster is reported.
(XLSX)

S3 Table PCA S7 Fig. Protein contribution values to the Principal component analysis (PCA) plot of Active, Inactive and Control samples based on the protein intensity detected in all analysed fractions (S7 Fig reported in S1 File).
(XLSX)

S4 Table Final cluster Exo annotations. The final protein sets from active/inactive CE and control samples were clustered together. The hierarchical dendrogram obtained was divided in 15 clusters on the basis of R coefficients. All protein clusters and their specific protein annotations are reported in the first sheet. In the following sheets tables and figures derived from the cluster information are reported.
(XLSX)

S5 Table Fraction 6. All information relative to the fraction most enriched in exosomes are reported. In the first sheet the resulting final clusters are reported, in the second sheet the protein contribution values to the PCA shown in Fig 5, and in the third sheet the protein-protein interaction enriched terms.
(XLSX)

S6 Table APhs-IPhs Dynet Networks. Active and inactive protein datasets (APhs and IPhs) were subjected to protein-protein interaction Dynet Network analysis. The network parameter values are reported for each protein in APhs or IPhs respectively in the first 2 sheets, then compared side by side in the third sheet and the fourth sheet reports the parameter values of the overlying Dynet graph.
(XLSX)

S7 Table APhs-IPhs String enrichments. The enriched GO Biological Process (first sheet) and Reactome Pathways (second sheet) identified in the APhs and IPhs are reported and compared.
(XLSX)
