# Supplementary material for: Proteomic analysis of plasma exosomes from Cystic Echinococcosis patients provides in vivo support for distinct immune response profiles in active vs inactive infection and suggests potential biomarkers
Source: PLoS Negl Trop Dis. 2020 Oct 5;14(10):e0008586. doi: 10.1371/journal.pntd.0008586 (PMC7535053; doi:10.1371/journal.pntd.0008586)

**Supporting Information**

**Protocol setup**

Plasma is a challenging fluid for proteomics, and for the purification of vesicles [43] due to the presence of highly abundant proteins, its viscosity and the presence of several kinds of lipid droplets, vesicles and protein aggregates. Several experiments were done using plasma pools from healthy people to evaluate the minimum volume and Relative Centrifugal Force to better separate MV from EXO. Blood from healthy people is known to present a lower concentration of circulating exosome and high variability [45]. We tested different starting volumes (from 5 to 100 ml), isolation strategies (UC: Ultracentrifugation; sucrose cushion; GD: sucrose density gradient; molecular weight cut-off); and Relative Centrifugal Force (RCF or g: gravity) for UC steps. To ensure the elimination of MV, we introduced between the 10K x g step, to eliminate cell debris and larger vesicles/organelles, and the 100K x g step, used to collect EXO, a further UC step at 30K x g. Thus, three sequential UC steps (S1A Fig), and GD resulted in a reduced yield but cleaner preparation. In our experience, to identify late-endosome ESCRT machinery or MVB markers (Vps28, Vps35, CHMP5) from plasma of healthy people (pools from 7 individuals) a large volume (100ml) of starting material was necessary. The SDS-PAGE of EXO and MV fractions 3-8 (density 1.05 – 1.21), the relative WB anti-CD81, and the PAP profiles of several proteins that were identified by MS analyses and which belong to the endocytosis pathway are shown in S1B, S2 Figs and S1 Table (Top3 abundance values are reported). However, to meet our real experimental conditions, i.e. sample collected from volunteers during a population screening, we reduced the plasma starting volume from 100 ml to 40 ml. Although the same protocol resulted in the loss of detection of some of the MVB and EXO markers, several were still detectable (S1C Fig and S1 Table). The presence of those markers also in the MV fractions, meaning a partial loss of EXO in MV pellet, was resolved reducing the RCF for the MV collection from 30K to 20K, and we finally obtained the results presented in Fig 2 and S1 Table.

S1 Fig


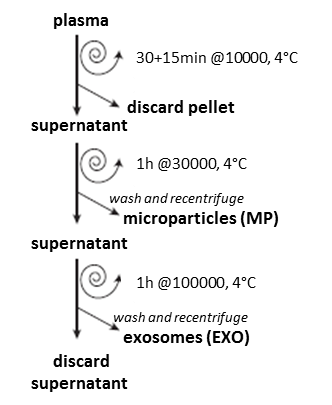


A


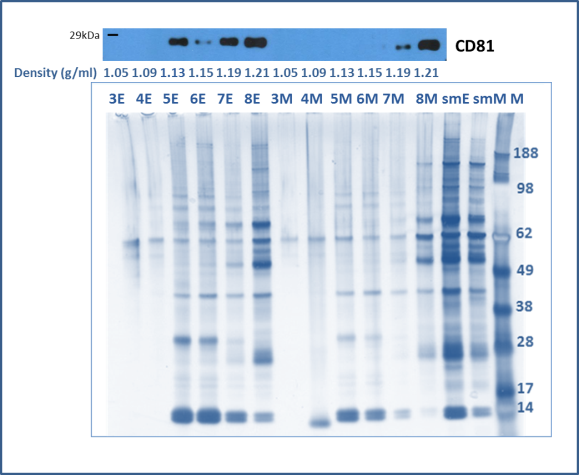


EXO MV


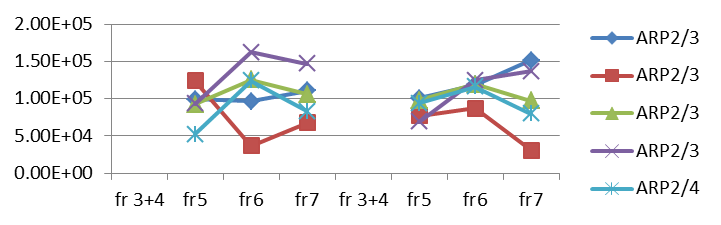

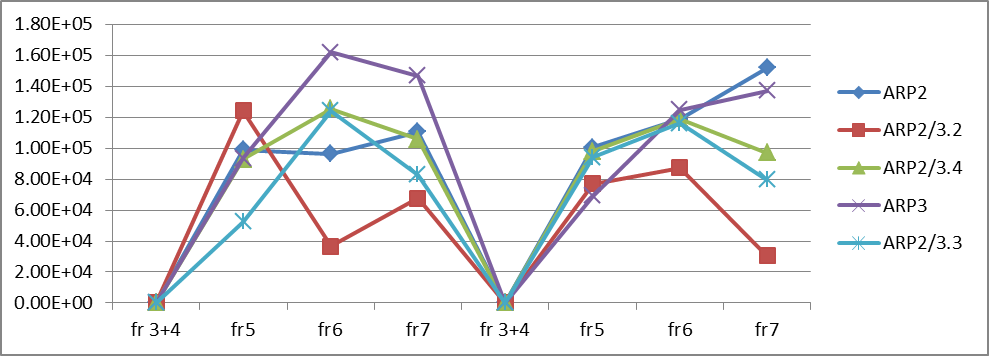

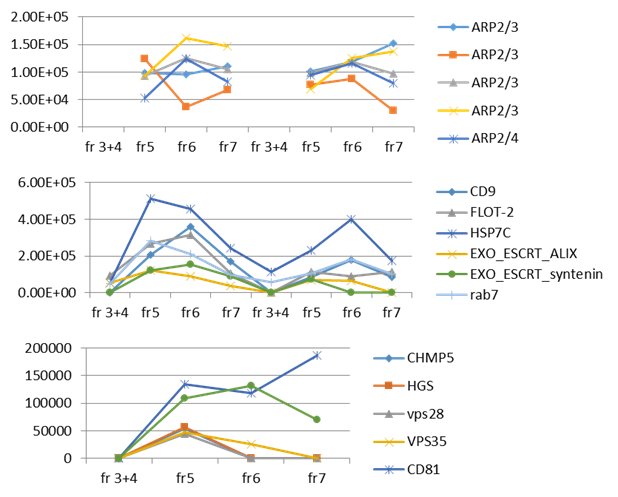


Clathrine coated vesicles

Late endosome and MVB

(±enriched)

MVB and exosomes

(found only in EXO fractions)

B


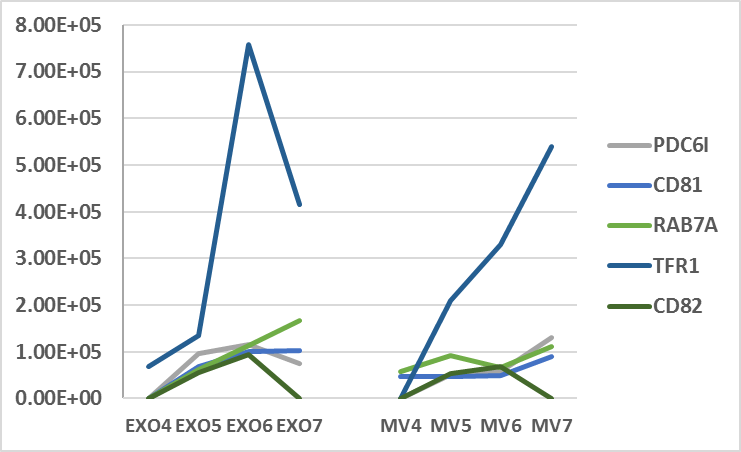


C

Intensity

Intensity

S2 Fig. KEGG Endocytosis pathway

The proteins identified in EXO preparation, obtained from a starting plasma volume of 100 ml, are coloured in pink.


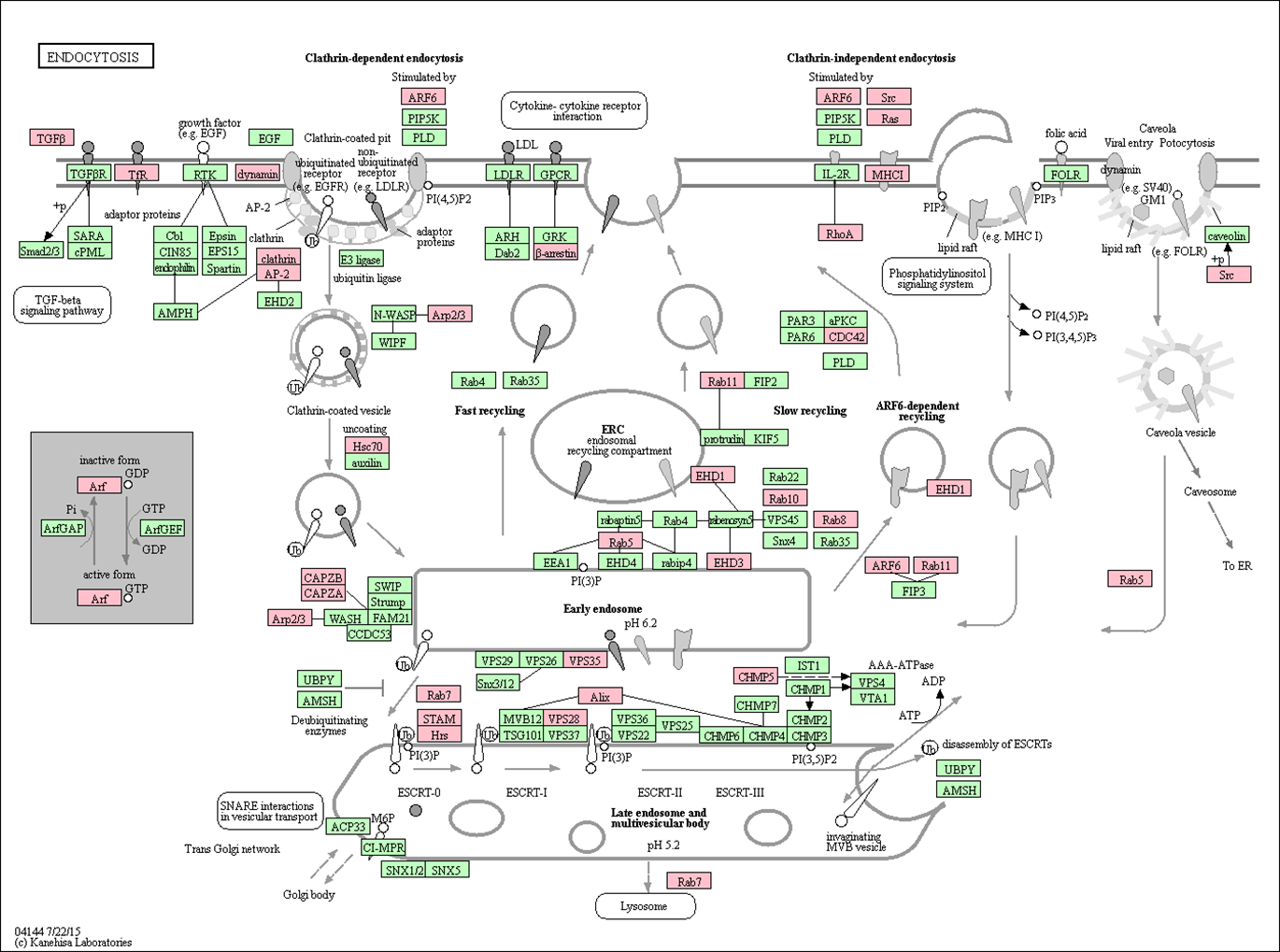


**Sample processing and normalization**

The four (or five fractions) to be analysed were chosen on the basis of their density values. S3 Fig reports the density values measured for all gradient fractions of samples analysed in this study by MS or WB as described in Table 1. Each chosen fraction (highlighted in yellow) was run on SDS-PAGE and each lane divided into 8 bands for in-gel digestion as shown in S4 Fig. The MS spectra of central fractions (generally fractions 5 and 6), mostly representative of exosome proteins, were merged for the construction of a three-point protein abundance profiles (PAP).

**S3 Fig**


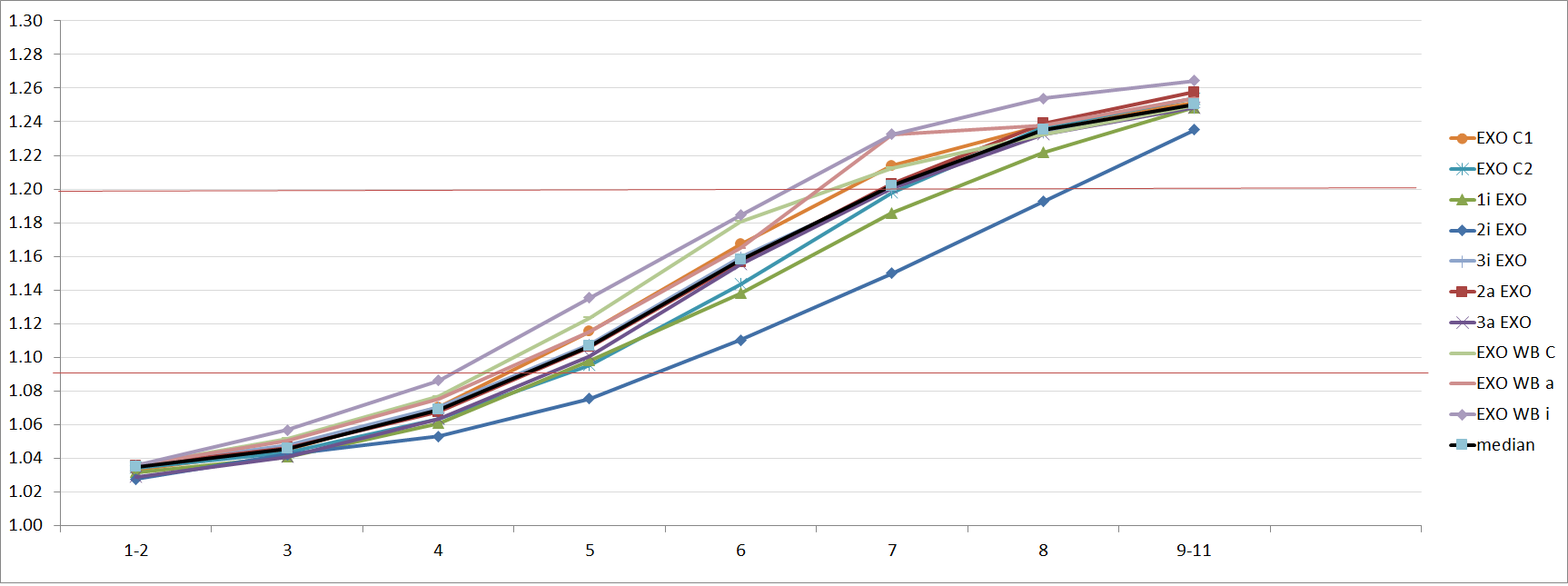


fraction

Besides measuring the source volume, normalization of EVs number in the case of biological fluids is not as easy as described for culture conditioned media (MISEV2018 and reference therein, [28]). The measurement of total protein amount or performing electron microscopy before purification are hampered by the abundance of circulating protein aggregates. Counting the number of EVs or quantifying the protein amount for each fraction would consume too much of the precious sample. Thus, before preparative SDS-PAGE for the LC-MS/MS we ran 1/10 of the sample on analytical PAGE for silver staining (cod. 246003, Pierce, Lifetech, 3 min of development) and performed a normalization quality control based on the evaluation of Hemoglobin, Albumin and IgGs contaminants (S4 Fig). We concluded that starting from the same volume of plasma (and in the case of controls also doubling it), the samples obtained for MS analyses were reasonably well normalized. On the base of the equivalent presence of those contaminant proteins, the qualitative and quantitative protein distribution over the gradient among the different samples would represent biological differences: different protein amount associated to exosomes, either for lower EXO amount or to lower protein cargo. The protein intensity of fraction C7 is indicative of higher concentration of contaminants in Ctr, likely because of a lower concentration of EXO.

S4 Fig


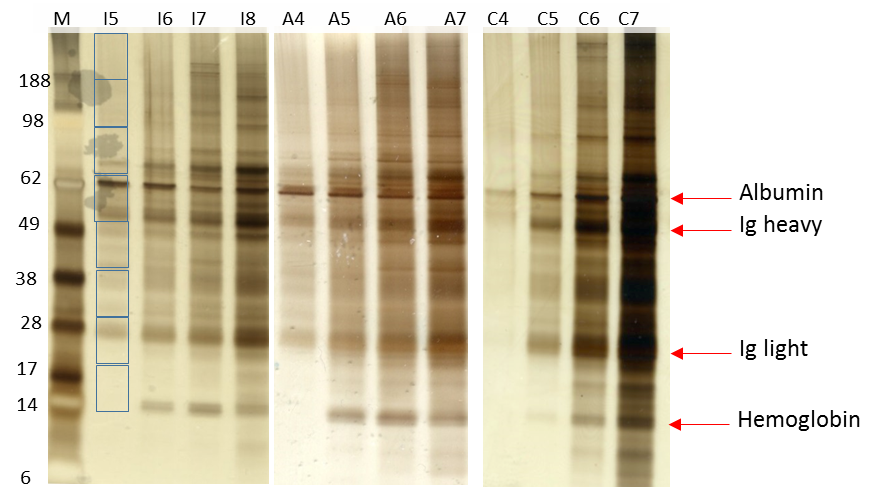


In cluster 2 of Fig 4A we found several non-EV co-isolated proteins which are often reported in EV studies. We reported in S5 Fig the PAP of some of those “contaminants” as additional evidence that samples were suitably normalized.

S5 Fig


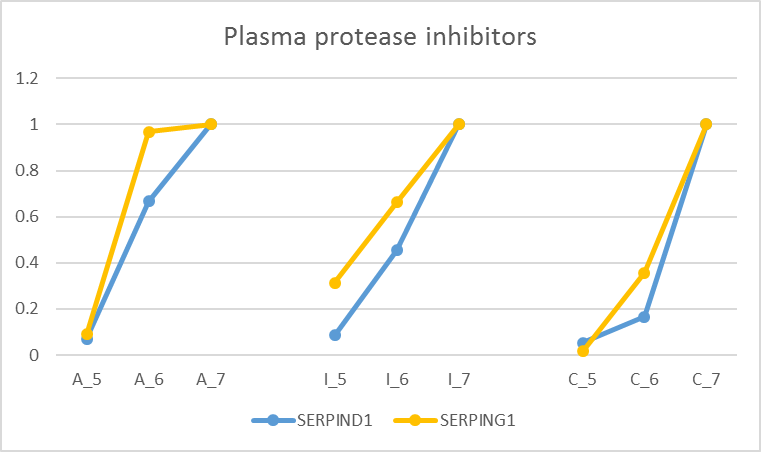


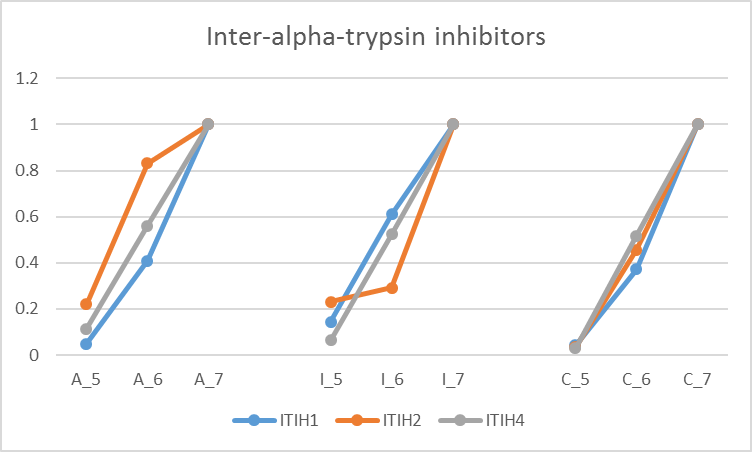


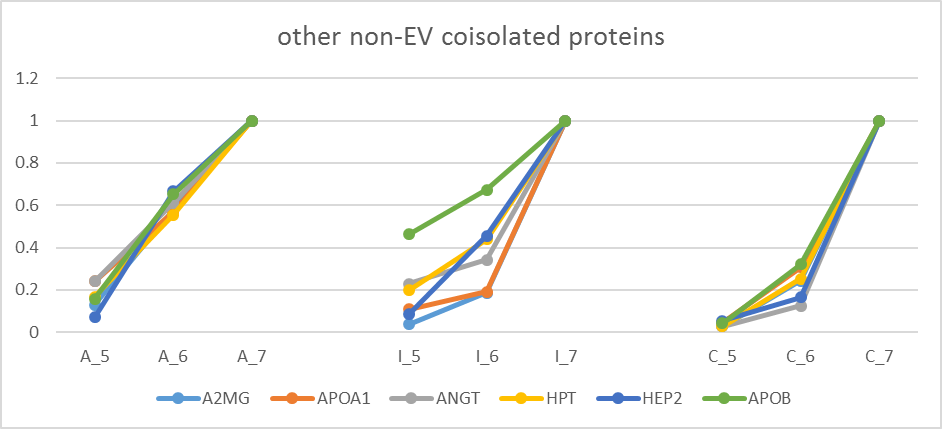


**Datasets definition and analysis**

S6 Fig. Hierarchical clustering of AP and IP proteins. Proteins identified in at least two replicas were submitted to hierarchical clustering (Cluster 3.0) and those nodes showing a PAP correlation R ≥ 0.7 (P ≤ 0.05) were selected for further analyses (red circles).


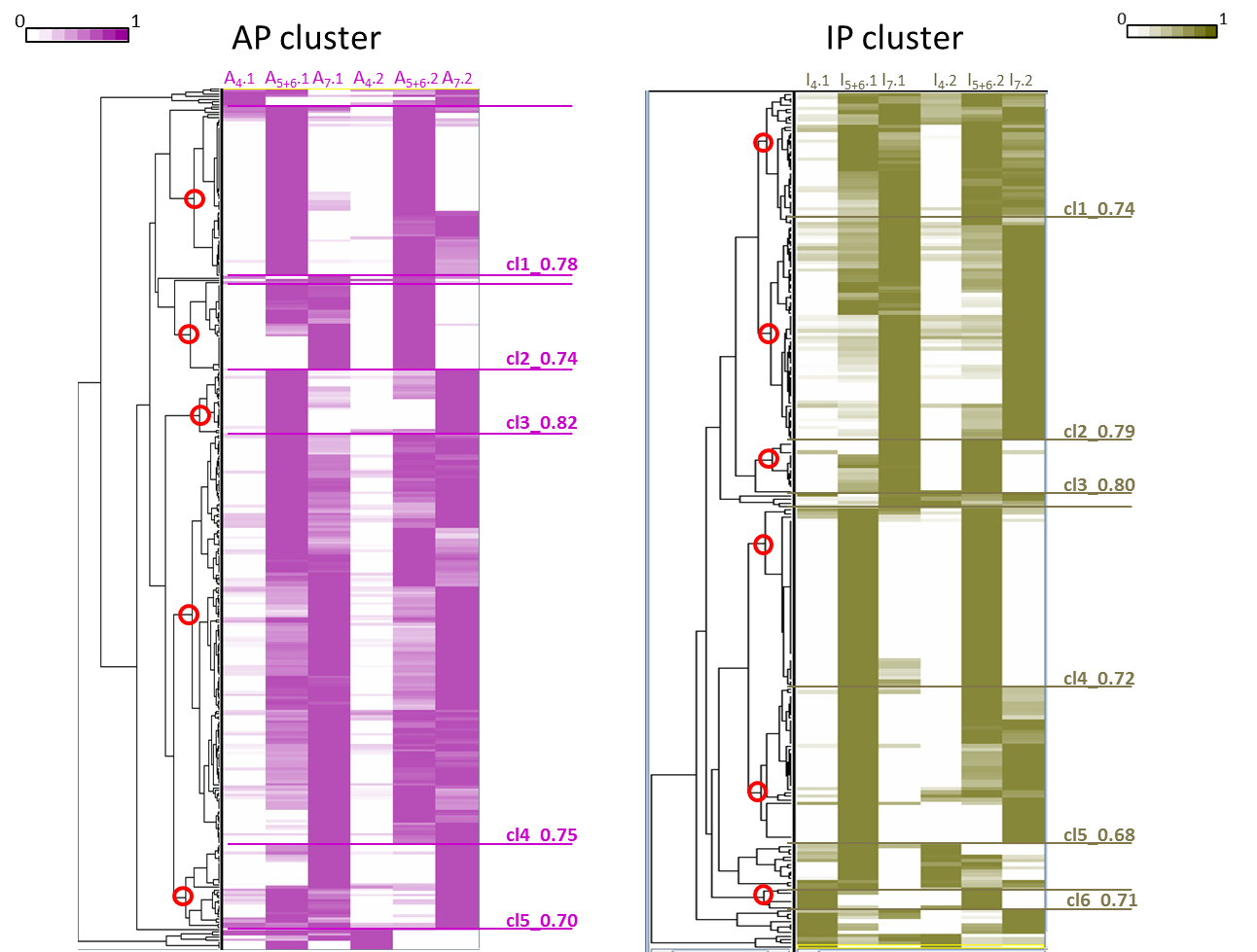


**S7 Fig.** Principal component analysis (PCA) plot of Active, Inactive and Control samples based on the protein intensity detected in all analysed fractions. The position of each replica for each sample in the two generated main components is plotted. Variability explained by the first and second components of the PCA is indicated between brackets. Single protein contributions are reported in S3 Table.

**Quality control of protein content-based EV characterization referred to recent bibliography**

S8 Fig shows the number of proteins representative of: the MISEV2018 [28] protein content-based EV characterization (numbered from 1 to 5); the proteins recognized to be involved in exosome release [49,50], and we extended this definition also to the *E. granulosus* (ECHGR) Rab-11a; the proteins most frequently identified in exosome studies (top 10/20/100 genes from Exocarta, [46]); the proteins already identified in CE-related EVs [51] and the remaining “other proteins”. For each category the percentage of the total dataset is reported.

S8 Fig


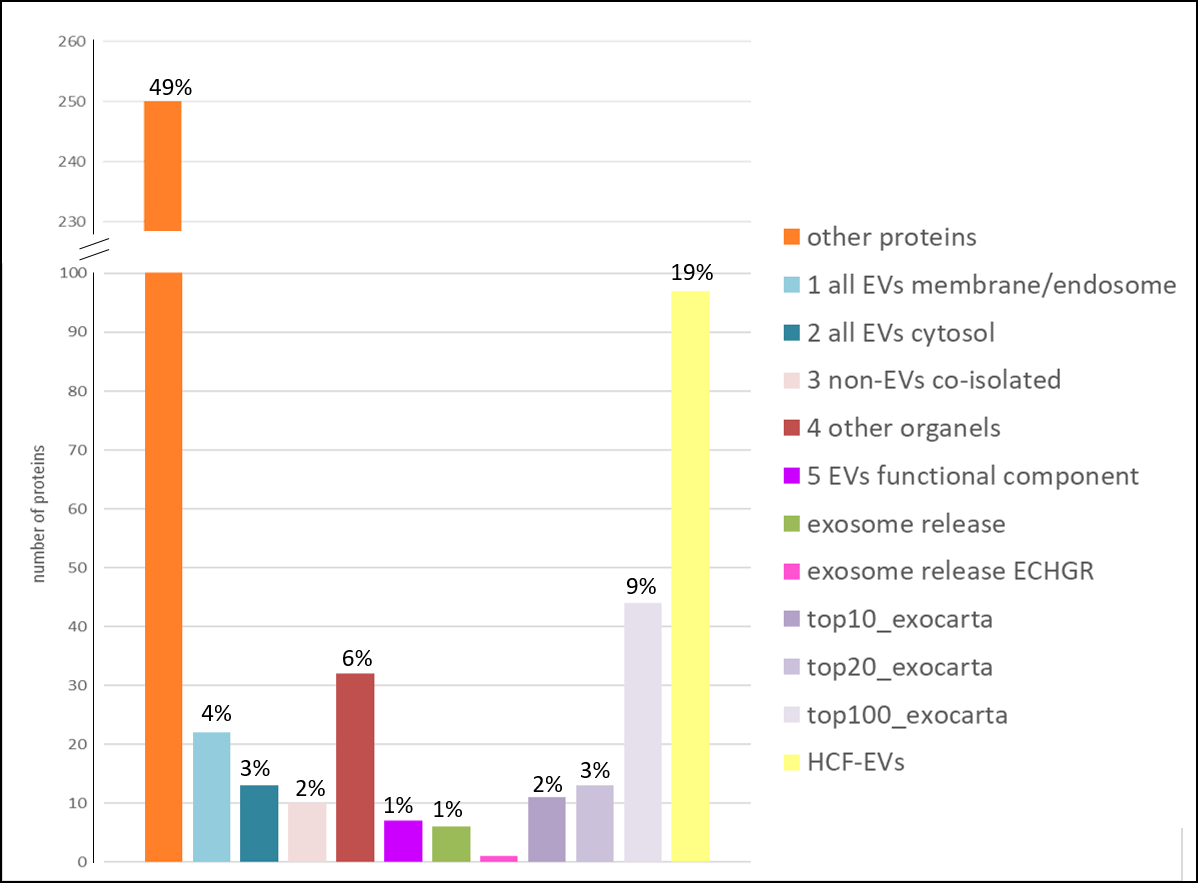

Supplement: S1 File — Supplementary information, figures and tables referred to exosome preparation and quality control of samples. (DOCX) [file pntd.0008586.s001.docx]
